# Vascular Smooth Muscle Cells Stimulate Platelets and Facilitate Thrombus Formation through Platelet CLEC-2: Implications in Atherothrombosis

**DOI:** 10.1371/journal.pone.0139357

**Published:** 2015-09-29

**Authors:** Osamu Inoue, Kazuya Hokamura, Toshiaki Shirai, Makoto Osada, Nagaharu Tsukiji, Kinta Hatakeyama, Kazuo Umemura, Yujiro Asada, Katsue Suzuki-Inoue, Yukio Ozaki

**Affiliations:** 1 Department of Clinical and Laboratory Medicine, Faculty of Medicine, University of Yamanashi, 1110 Shimokato, Chuo, Yamanashi 409–3898, Japan; 2 Department of Pharmacology, Hamamatsu University School of Medicine, Shizuoka, Japan; 3 Department of Pathology, Faculty of Medicine, University of Miyazaki, Miyazaki, Japan; University of Iowa, UNITED STATES

## Abstract

The platelet receptor CLEC-2 is involved in thrombosis/hemostasis, but its ligand, podoplanin, is expressed only in advanced atherosclerotic lesions. We investigated CLEC-2 ligands in vessel walls. Recombinant CLEC-2 bound to early atherosclerotic lesions and normal arterial walls, co-localizing with vascular smooth muscle cells (VSMCs). Flow cytometry and immunocytochemistry showed that recombinant CLEC-2, but not an anti-podoplanin antibody, bound to VSMCs, suggesting that CLEC-2 ligands other than podoplanin are present in VSMCs. VSMCs stimulated platelet granule release and supported thrombus formation under flow, dependent on CLEC-2. The time to occlusion in a FeCl_3_-induced animal thrombosis model was significantly prolonged in the absence of CLEC-2. Because the internal elastic lamina was lacerated in our FeCl_3_-induced model, we assume that the interaction between CLEC-2 and its ligands in VSMCs induces thrombus formation. Protein arrays and Biacore analysis were used to identify S100A13 as a CLEC-2 ligand in VSMCs. However, S100A13 is not responsible for the above-described VSMC-induced platelet activation, because S100A13 is not expressed on the surface of normal VSMCs. S100A13 was released upon oxidative stress and expressed in the luminal area of atherosclerotic lesions. Suspended S100A13 did not activate platelets, but immobilized S100A13 significantly increased thrombus formation on collagen-coated surfaces. Taken together, we proposed that VSMCs stimulate platelets through CLEC-2, possibly leading to thrombus formation after plaque erosion and stent implantation, where VSMCs are exposed to blood flow. Furthermore, we identified S100A13 as one of the ligands on VSMCs.

## Introduction

CLEC-2 has been identified as a receptor for the platelet-activating snake venom rhodocytin/aggretin[1]. It elicits robust platelet aggregation through a tyrosine kinase-depending signaling pathway[1]. We identified podoplanin as an endogenous ligand for CLEC-2 for the first time[2]. Podoplanin is expressed on the surface of tumor cells and induces platelet aggregation by binding to CLEC-2, facilitating hematogenous tumor metastasis[2, 3]. It is also expressed in lymphatic endothelial cells, but not in vascular endothelial cells[4]. CLEC-2-deficient mice show embryonic/neonatal lethality and blood-filled lymphatic vessels[5–7], suggesting that CLEC-2 facilitates blood/lymphatic vessel separation by interacting with podoplanin in lymphatic endothelial cells[8, 9].

Three studies reported that CLEC-2 plays a role in thrombosis and hemostasis[6, 10, 11], and one negative report has also been published[7]. May et al. showed that CLEC-2 is an essential platelet-activating receptor in hemostasis and thrombosis, using mice with anti-CLEC-2 antibody-induced CLEC-2 deficiency[10]. Moreover, by transplantation of fetal liver cells from CLEC-2^+/+^ or CLEC-2^−/−^ embryos, we demonstrated that CLEC-2 is involved in thrombus stabilization *in vitro* and *in vivo*, possibly through homophilic interactions between pairs of CLEC-2 molecules, without an apparent increase in bleeding tendency[6]. In contrast, Hughes et al. showed that CLEC-2 is not required for platelet aggregation under arteriolar shear in an *in vitro* flow system[7]. However, it has recently been reported that combined *in vivo* depletion of the collagen receptor glycoprotein (GP) VI and CLEC-2 severely compromises hemostasis and abrogates arterial thrombosis in mice[11], indicating that CLEC-2 plays a role in thrombosis and hemostasis, although deletion of CLEC-2 alone produces a relatively minor phenotype. A tendency for severe bleeding in the absence of both receptors leads us to speculate that, along with CLEC-2, ligands for CLEC-2 are present in vessel walls (homophilic association). Therefore, identifying an endogenous ligand for CLEC-2 in vessel walls that contributes to thrombosis and hemostasis is important, since it may facilitate development of treatments for thrombosis and hemostasis.

In the present study, we demonstrated that VSMCs stimulate platelets through binding between CLEC-2 and its ligands. We identified S100A13 as one of the ligands, but founds that it has limited potency; the other ligand remains unknown. Both may contribute to thrombus formation after plaque erosion and stent implantation, when VSMCs are exposed to blood flow. CLEC-2 ligands other than podoplanin are expressed on the surface of vascular smooth muscle cells (VSMCs) and that association between CLEC-2 and the ligand activates platelets and facilitates thrombus formation under flow conditions. We speculate that this association plays a pathophysiological role in thrombus formation after stent thrombosis and plaque erosion, when VSMCs are most likely to be exposed to blood flow. We identified S100A13 as the CLEC-2 ligand in VSMCs, although its physiological significance remains to be clarified.

## Materials and Methods

### Materials

An EnVision kit (rabbit IgG), Anti-α-smooth muscle actin (SMA; clone 1A4), and anti-CD68 (clone PGM-1) were purchased from Dako. The recombinant extracellular domain of human (h) or mouse (m) CLEC-2 was expressed as a dimeric human (h), rabbit (r), or rat immunoglobulin Fc domain fusion protein (hCLEC-2-hFc2, hCLEC-2-rFc2, or mouse CLEC-2-ratFc2); rFc2, hFc2, and ratFc2 were generated as described previously[2]. The GPVI agonist collagen-related peptide (CRP) was generated as previously described[12] by the Peptide Institute (Japan). Rhodocytin was generated as described previously[13]. GST-tagged S100A13 recombinant protein was purchased from CycLex Co., Ltd. (Japan) and His-tagged S100A13 recombinant protein was purchased from R&D Systems Inc. GST-tagged S100A13 recombinant protein was used for experiments unless otherwise noted. Other reagents were obtained from sources described below or previously described[6, 9].

### *In situ* binding assay and immunohistochemistry

We used human abdominal aorta from a patient autopsied at the University of Miyazaki Hospital in 2001 after written informed consent was provided by the family. The Ethics Committees of the University of Miyazaki approved the study protocol (No.942) and the study was performed in accordance with the ethical standards of the Declaration of Helsinki. Several fresh aortic tissues (2 cm × 2 cm) were collected from atherosclerotic lesions of various degrees, and the specimens were frozen in Optimal Cutting Temperature (OCT) compound. Serial sections of the aortic intima (4 μm thick) were immunohistochemically stained with the indicated primary antibodies or recombinant proteins, as described previously using the EnVision kit [14, 15].

For the binding assay for the mouse aorta and lung tissue, perfusion fixation of 12-week-old C57BL/6 mice with 4% PFA/PBS was performed, and the abdominal aorta and lungs were harvested and fixed with 4% PFA/PBS for 2 h at 4°C. After washing in PBS, the tissues were equilibrated in 30% sucrose in PBS, embedded in OCT compound, and sectioned at 10-μm thickness. The sections were washed in PBS and blocked with 5% skim milk/PBS for 1 h. Each specimen was incubated with mCLEC-2-ratFc2 (25 μg/mL), rat Fc2 as a control (25 μg/mL), or anti-smooth muscle actin (Thermo Scientific) overnight at 4°C. Color detection was performed using Histofine Simple Stain MAX PO (rat and rabbit) and Histofine DAB-3S kit (Nichirei Biosciences, Japan) following the manufacturer’s protocol. For podoplanin staining, each specimen was incubated with an anti-mouse podoplanin antibody (clone 8.1.1; Santa Cruz Biotechnology, Inc.) or control hamster IgG (Santa Cruz Biotechnology, Inc.). After rinsing with PBS, the samples were stained with anti-hamster IgG labeled with Alexa Fluor 488 (Life Technologies Inc.) for 2 h at 4°C. After rinsing with PBS, the cells were observed using an inverted fluorescence microscope IX71 (Olympus, Japan).

Where indicated, serial frozen-thawed sections of the mouse abdominal aorta from wild-type or ApoE-deficient mice fed a high fat diet were incubated with control rabbit IgG, anti-smooth muscle actin (Thermo Scientific, #RB-9010-P0), or anti-S100A13 antibody, followed by anti-rabbit IgG-Alexa Fluor 488. For co-staining for smooth muscle actin and S100A13, we used anti-α smooth muscle actin antibody (Abcam, ab21027), followed by anti-goat IgG Alexa Flour 546 (Life Technologies Inc.). The sections were counter-stained with 4',6-diamidino-2-phenylindole (DAPI). The images are visualized a laser confocal microscope, FluoView (Olympus).

### Cells

Human coronary artery smooth muscle cells (CASMCs) were purchased from Lonza and maintained on culture dishes in smooth muscle cell basic medium (SmBM) supplemented with SmGM-2 SingleQuots media (Lonza). 293TREx cells were obtained from Life Technologies Inc. Cultures were maintained at 37°C, 5% CO_2_, and 100% humidity. To generate suspended CASMCs, we washed immobilized CASMCs in 10 cm culture dishes with PBS once, added 2 mL of trypsin/EDTA (0.025% trypsin, 0.02% EDTA), removed the trypsin/EDTA, incubated the dishes at 37°C for 2 min, and resuspended with PBS. After counting the number of cells, we centrifuged the suspended CASMCs at 180 g for 5 min, removed the supernatant, and resuspended the cells with PBS at the indicated cell concentrations.

Where indicated, CASMCs were cultured with 10% fetal bovine serum-containing Dulbecco's Modified Eagle's Medium (DMEM), with or without 0.5 mM, 1 mM, or 2 mM hydrogen peroxide (H_2_O_2_) for 2 d, as previously described[16]. The H_2_O_2_-treated CASMCs were washed with PBS once, incubated with 2 mL of trypsin/EDTA for 2 min at 37°C, and neutralized by addition of 4 mL of completed DMEM. The CASMCs were washed and suspended at 5 × 10^6^/mL in PBS containing 0.1 mM CaCl_2_, as described above.

### Flow cytometry studies

Cells suspended in PBS (5 × 10^6^/mL, 100 μL) were incubated with the primary antibodies control IgG or anti-podoplanin Ab (NZ-1) at 5–10 μg/mL or the recombinant proteins (hFc2 or hCLEC-2-hFc2) at 100 ∞g/mL. After washing with PBS, these cells were resuspended with 100 ∞L of PBS and stained with 3 ∞L of secondary antibodies (anti-hamster IgG or anti-human IgG) conjugated with fluorescein isothiocyanate (FITC) for 15 min.

Where indicated, cells suspended in modified Tyrode’s buffer supplemented with vehicle, 1 mM CaCl_2_, or 1 mM EDTA were incubated with or without GST-tagged S100A13 recombinant protein at 20 ∞g/mL. Then the unbound recombinant proteins were washed with PBS and the binding of S100A13 protein to the cells was detected by 10 ∞g/mL anti-S100A13 antibody (63Y, Santa Cruz Biotechnology, Inc.) and anti-mouse IgG conjugated with Alexa Fluor 488.

Surface expression of endogenous S100A13 was also analyzed by flow cytometry. CASMCs pretreated with or without H_2_O_2_ as described above were incubated with 10 ∞g/mL control mouse IgG or anti-S100A13 antibody (63Y), followed by Alexa Fluor 488-conjugated anti-mouse IgG.

The stained cells were immediately analyzed using a FACScan or BD Accuri (BD Biosciences). The data were recorded and analyzed using CellQuest software or BD Accuri CFlow software.

### Immunocytochemistry

CASMCs or 293TREx cells were cultivated on 24-well culture dishes. After rinsing with PBS, CASMCs were incubated with recombinant rabbit Fc2 (100 ∞g/mL), recombinant human CLEC-2 rabbit Fc2 (100 ∞g/mL), or anti-podoplanin antibody (NZ-1, 1:50) for 2 h at 4°C. After rinsing with PBS, CASMCs were stained with goat anti-rabbit IgG labeled with Alexa Fluor 488 (1:200) for 2 h at 4°C. After rinsing with PBS, cells were observed using an inverted fluorescence microscope IX71 (Olympus).

For S100A13 staining for CASMCs, CASMCs were cultivated on 24-well culture dishes. After rinsing with PBS, CASMCs were fixed with 3% paraformaldehyde for 30 min and then incubated with control mouse IgG or anti-S100A13 antibody (63Y) for 30 min at 37°C. Where indicated, the fixed cells were permeabilized by 0.1% Triton X-100 for 5 min. After rinsing with PBS, CASMCs were stained with anti-mouse IgG labeled with Alexa Fluor 488 (1:200) for 30 min at 37°C. After rinsing with PBS, cells were observed using an inverted fluorescence microscope IX71 (Olympus).

### Animals

Because CLEC-2-null mice are embryonic/neonatal lethal, CLEC-2-deficient irradiated chimeric mice were generated as described previously[6]. Briefly, irradiated adult C57BL/6 male mice were rescued by intravenous injection of 1 × 10^6^ fetal liver cells from CLEC-2^−/−^ (CLEC-2-deficient chimera) or CLEC-2^+/+^ embryos (wild-type (WT) chimera). We confirmed complete bone marrow engraftment in all the CLEC-2-deficinet chimeras by flow cytometry. C57Bl/6-Tg (CAG-EGFP) mice were purchased from Japan SLC Inc. These mice were crossed with CLEC-2^+/−^ mice to generate CAG-EGFP;CLEC-2^+/−^ offspring. CAG-EGFP;CLEC-2^+/−^ mice were crossed to obtain CAG-EGFP;CLEC-2^+/+^ and CAG-EGFP;CLEC-2^−/−^ embryos. Where indicated, these GFP-positive embryos were used to generate GFP-positive WT chimeras and GFP-positive CLEC-2-deficient chimeras.

Apolipoprotein (Apo) E-/- mice were purchased from Jackson Laboratories and fed a high fat diet for 8 weeks. This study was approved by the Animal Care and Use Committee at the University of Yamanashi.

### Platelet Preparation

For preparation of mouse washed platelets, the WT chimeras or CLEC-2-deficient chimeras were killed with diethyl ether, and blood drawn by post-caval puncture was collected into 100 ∞L of acid citrate-dextrose. The washed murine platelets were obtained through centrifugation, as previously described, using prostacyclin to prevent activation during the isolation procedure[17]. Platelets were resuspended in the modified Tyrode’s buffer[17] at the indicated cell densities. The reconstituted blood included washed platelets and washed RBCs (6 × 10^8^/mL platelets and 4 × 10^6^/∞L RBCs). RBCs were washed twice using RBC washing buffer (140 mM NaCl, 10 mM HEPES, 5 mM glucose, pH 7.2) and resuspended in the RBC washing buffer containing 0.04 U/mL apyrase and 0.1 mg/mL D-Phe-Pro-Arg-chloromethylketone (PPACK).

For preparation of human platelets, venous blood from healthy drug-free volunteers was collected into 10% sodium citrate. Platelet-rich plasma (PRP) was obtained after centrifugation of whole blood at 160 × *g* for 10 minutes. The platelets were washed twice with a buffer containing 15% acid-citrate dextrose and 100 nmol/L PGI_2_ and then resuspended in modified Tyrode’s buffer at a cell density of 1 × 10^9^/mL. This study was approved by the Ethical Committee at the University of Yamanashi, and written informed consent was provided according to the Declaration of Helsinki.

### Platelet aggregation

Platelet aggregation by CHO cells transfected with human podoplanin or CASMCs was investigated as described previously[2]. 1.5 or 6 ∞L of the cells at 5 × 10^7^ cells/ml was added to human washed platelets (300 ∞L, 2 × 10^9^/mL). Final concentrations of the cells were 2.5 × 10^5^ cells/ml and 1.0 × 10^6^ cells/ml. Platelet aggregation was monitored by measuring light transmission with the use of an AG-10 aggregation analyzer (Kowa, Tokyo, Japan) for 10 min under constant stirring at 1000 rpm at 37°C. The instrument was calibrated with buffer for 100% transmission.

### Measurement of platelet granule release reaction

Washed platelets (200 ∞L of 4 × 10^8^/mL) obtained from the CLEC-2-deficient chimeras were incubated with 200 ∞L of modified Tyrode’s buffer, 200 μL of CASMCs (5 × 10^6^/mL in modified Tyrode’s buffer), 200 ∞L of 1 μg/mL CRP, 200 μL of 100 nM rhodocytin, or 200 μL of lysis buffer for 10 min. Then, platelets were centrifuged at 1100 g for 8 min in the presence of 1 μg/mL PGI_2_, and the supernatants were collected into new tubes. The amount of secreted 5-HT or PDGF was measured using a serotonin-ELISA kit (DLD Diagnostika GmbH) and a Human/Mouse PDGF-AA Quantikine ELISA kit (R&D Systems Inc.), respectively, according to the manufacturer’s instructions. Platelet lysates were used to measure the total amount of 5-HT or PDGF stored in the platelets. The results were expressed as percentages of secreted 5-HT or PDGF with respect to the total amounts stored in the platelets.

### Flow adhesion assay

CASMCs were cultivated in a cell microscopy chamber (1 microSlide VI0.1 ibiTreate; Ibidi) and blocked with SmBM culture medium containing 0.5% BSA for 1 h. Where indicated, capillary tubes (0.3 mm × 1.2 mm, 50 mm long) were coated overnight at 4°C with PBS or 100 μg/mL collagen and blocked with 5% BSA. Platelet concentrations were counted 1 week before the experiments to equalize platelet counts between WT and CLEC-2 chimeras. GFP-positive WT chimeras, GFP-positive CLEC-2-deficient chimeras, CLEC-2-deficient chimeras, or WT chimeras were killed using diethyl ether. Murine blood was drawn via a post-caval puncture and anti-coagulated using 40 μM PPACK and 5 units/mL heparin. Murine blood from CLEC-2-deficient chimeras or WT chimeras was pretreated with 5 μM 3-dihexyloxacarbocyanine iodide (DiOC6) for 30 min. After the chamber was rinsed with modified Tyrode’s buffer supplemented with 2 mM CaCl_2_ and 1 unit/mL heparin, the blood sample was perfused into the chamber for 5 min at a shear rate of 400 s^−1^. Adherent platelets were visualized using a fluorescence video microscope. Movie data were converted into sequential photo images. The zero min images were captured just before perfusion under bright field illumination. After 5 min of perfusion, the blood was gently washed out using PBS and adherent platelets were fixed in 3% PFA for 10 min. For measurement of thrombus volume, adherent platelets were visualized using a FV-1000 confocal laser microscope and z-stack imaging was performed according to the manufacturer’s instructions. The z-stack data were analyzed using FluoView (Olympus). The integrated fluorescence intensity of GFP and the background signal were calculated, and the background signal was subtracted from the integrated fluorescence intensity of GFP (corrected IFI; cIFI). The thrombus volume was expressed as the cIFI per image (404374 μm^2^).

Where indicated, capillary tubes (0.3 mm × 1.2 mm, 50 mm long) were coated overnight at 4°C with 50 μg/mL collagen or 50 μg/mL collagen plus 50 μg/mL GST-tagged S100A13. Capillaries were washed and blocked with PBS containing 2% BSA for 2 h at room temperature. They were then rinsed with modified Tyrode’s buffer supplemented with 2 mM CaCl_2_ and 1 unit/mL heparin. Human venous blood from healthy drug-free volunteers was collected into 0.1 mg/ml argatroban (a synthetic thrombin inhibitor) and 5 units/mL heparin. The human or murine blood was pretreated with 5 μM DiOC6 for 30 min and then perfused into the capillaries at 1500 s^-1^. After 10 min or 5 min (murine blood) of perfusion, thrombus formation was visualized and quantified as described above.

### *In vivo* thrombosis model

Platelet concentrations were counted 1 week before the experiments to equalize platelet counts between WT and CLEC-2 chimeras. The WT chimeras or CLEC-2-deficient chimeras were anesthetized by inhalation of 4% halothane and maintained under anesthesia with 2% halothane. Body temperature was monitored using a rectal probe and maintained at 37°C on a heat pack. Anesthetized mice were secured in the supine position under a surgical microscope (KONAN, Japan). The femoral artery was exposed by blunt dissection. For photochemically induced thrombosis (PIT), vascular injury was photochemically induced in the mouse femoral artery, as described previously[18]. In brief, a cannula was inserted into the jugular vein of the anesthetized mice for injection of rose Bengal. The right femoral artery was carefully exposed, and the probe of a Doppler blood flow velocimeter (Cristal Biotec Inc.) was attached to the branch point of the deep femoral artery distal to the inguinal ligament for monitoring blood flow. Transillumination of the exposed segment with green light (540 nm) was achieved using a xenon lamp with a heat-absorbing filter and a green filter (Hamamatsu Photonics). The use of a heat-absorbing filter prevented excessive generation of heat capable of damaging biological tissues. The irradiation was directed using a fiber-optic filament positioned 2 mm away from a segment of the intact femoral artery proximal to the flow probe. Irradiation at an intensity of 0.9 W/cm^2^ was started after baseline blood flow had stabilized. Five minutes later, rose Bengal (20 mg/kg) was infused for 5 min. The irradiated segment of the femoral artery was considered to be occluded when blood flow had completely stopped. The experimental protocol in this study was approved by the Hamamatsu University Committee on Ethics of Animal Experimentation. For the FeCl_3_ model, a 1 mm × 1 mm strip of filter paper saturated with 10% FeCl_3_ (Sigma-Aldrich) solution was applied to the adventitial surface of the exposed femoral artery for 3 min. Femoral blood flow was monitored using a Doppler blood flow velocimeter (Cristal Biotec, or Advance Co. Ltd., Japan) for 30 min. The irradiated segment of the femoral artery was considered to be occluded when blood flow had completely stopped. The experimental protocol in this study was approved by the Hamamatsu University and the University of Yamanashi Committee on Ethics of Animal Experimentation.

### Victoria blue–hematoxylin-eosin (HE) staining

After confirming vessel occlusion, the uninjured- and photochemically-injured or FeCl_3_-injured femoral arteries were removed from euthanized mice and fixed in 3.7% formalin and embedded in paraffin. Five-micron sections were stained with Victoria blue (Wako, Japan), followed by HE staining.

### Protein Array

Protein array was performed using ProtoArray® Human Protein Microarray (Life Technologies Inc.), according to the manufacturer’s protocol.

### Surface plasmon resonance spectroscopy

hCLEC-2-rFc2 and rFc2 were covalently coupled to a CM5 chip (Biacore AB) using an amine coupling kit according to the manufacturer’s instructions[19]. The specific interaction between hCLEC-2-rFc2 and recombinant S100A13 was analyzed using a Biacore X system (Biacore AB). Recombinant S100A13 proteins (GST-tagged or His-tagged) in modified Tyrode’s buffer supplemented with 1 mM CaCl_2_ were perfused over the control surface or an immobilized hCLEC-2-rFc2 surface at a flow rate of 20 μL/min at 25°C; changes in the resonance were recorded. The response from the hCLEC-2-rFc2 surface was subtracted from that of the control surface. Regeneration of the protein-coated surfaces was achieved by running 10 mM HCl through the flow cell at 20 μL/min, twice. The dissociation constants (Kd) were determined using BIAevaluation software.

### Western blotting

CASMCs (5 × 10^6^/mL), washed human platelets (1 × 10^9^/mL), or recombinant His-tagged S100A13 protein (1 μg/mL) were separated by electrophoresis[9] and western blotted with rabbit monoclonal anti-S100A13 antibody (Epitomics) or anti-β3 integrin antibody (Clone 1, BD Biosciences).

### Platelet adhesion assay

Coverslips were coated with 250 μg/mL of collagen and 100 μg/mL of recombinant S100A13-GST and maintained overnight at 4°C. After washing twice with PBS, the coverslips were blocked with 1% fatty acid-free BSA in PBS for 2 h. BSA-coated coverslips were prepared as negative controls. Washed murine platelets (6 × 10^7^/mL) were seeded on the coverslips for 30 min at room temperature in the presence or absence of 1 mM EDTA. Adherent platelets were stained with TRITC-conjugated phalloidin as described previously[20]. Platelets were visualized using an inverted fluorescence microscope (IX71) equipped with a 100× or 60× objective lens, a monochromatic light source, and a DP-73 digital camera (Olympus). Five to 10 images from two independent experiments were photographed using a 60× objective lens and randomly selected per experiment. Adherent platelets were counted (0.022 mm^2^/image) under blind conditions. Images were photographed with a 100× objective lens.

### Statistics

Statistical significance was evaluated using the Mann–Whitney U test, the Welch’s t test, or the Student’s t-test. In each case, p-values < 0.05 were considered to be statistically significant.

## Results

### Recombinant CLEC-2 bound to early and advanced atherosclerotic lesions

**Fig 1:**
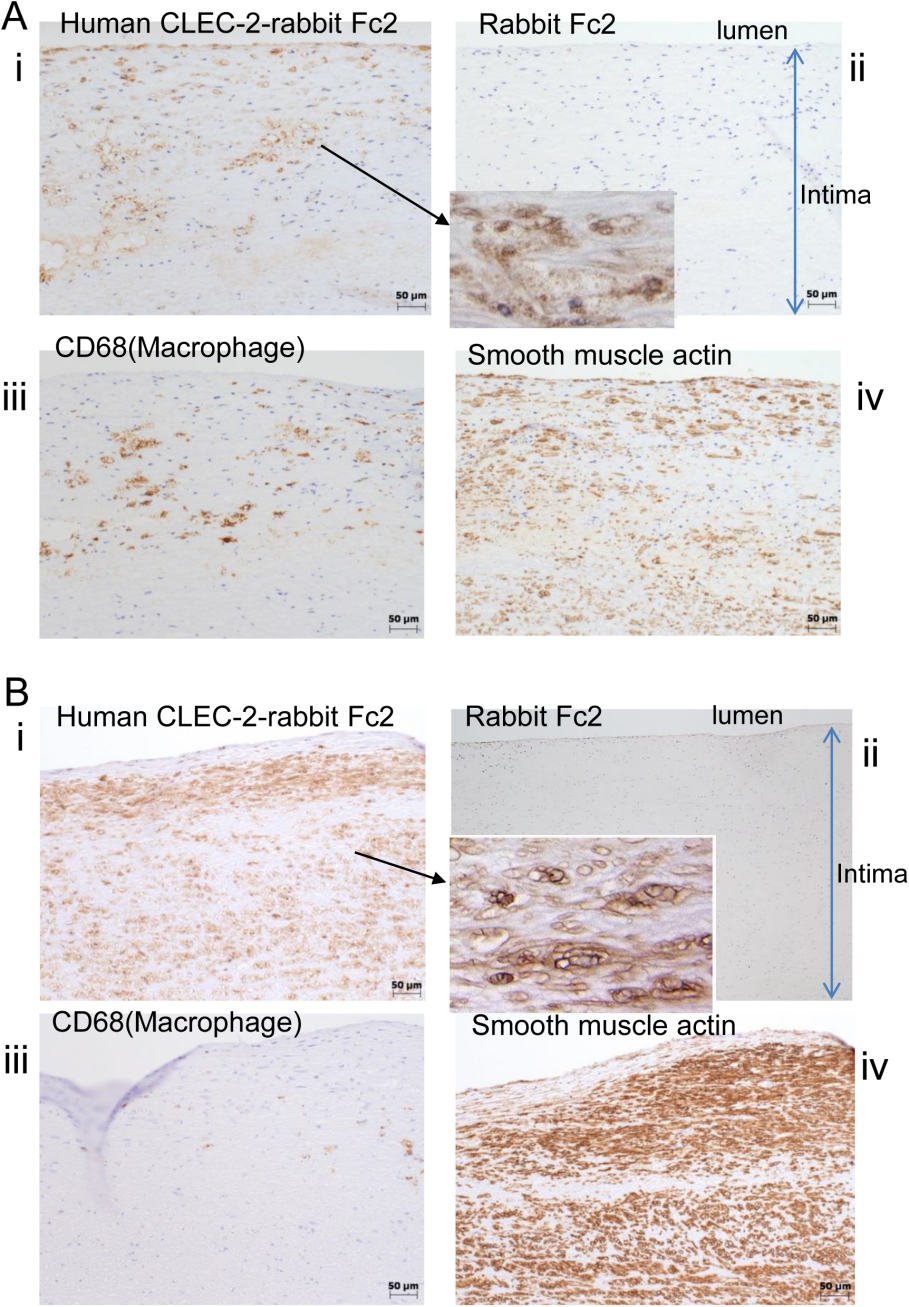
Recombinant CLEC-2 bound to early and advanced atherosclerotic lesions. A) Advanced lesions (fibrofatty plaques) of the human abdominal aorta were stained by (i) human CLEC-2-rabbit Fc2, (ii) rabbit Fc2, (iii) anti-CD68 (clone PGM-1), and iv) anti-smooth muscle actin. Magnified Fig 1A(i) was inserted into (ii). B) Early lesions (diffuse intimal thickening) were stained by (i) human CLEC-2-rabbit Fc2, (ii) rabbit Fc2, (iii) anti-CD68 (clone PGM-1), and iv) anti-smooth muscle actin. Magnified Fig 1Bi was inserted into (ii).

To investigate whether a CLEC-2 ligand(s) is present in the vessel wall, we generated a recombinant human CLEC-2 extracellular domain expressed as a dimeric human immunoglobulin Fc domain fusion protein (hCLEC-2-rFc2). Then, we examined whether hCLEC-2-rFc2 binds to the wall of a human abdominal aorta obtained from an autopsied patient. Atherosclerotic lesions were categorized histologically into three types, i.e., two types of early lesions (diffuse intimal thickening (DIT) and fatty streak) and one type of advanced lesion, according to the American Heart Association classification[21]. We chose to examine two of these lesion types: DIT and advanced lesions. DIT, which is considered to be virtually normal intima, is composed exclusively of proliferated smooth muscle cells and extracellular matrix. Advanced lesions include atheromatous plaques, which contain proliferated smooth muscle cells, lipid-laden macrophages, a large amount of extracellular matrix, with a central necrotic core. hCLEC-2-rFc2 bound to advanced lesions of the autopsied aorta (Fig 1Ai), whereas no binding of control rFc2 recombinant proteins was detected (Fig 1Aii). The advanced lesions were rich in macrophages, which stained with CD68 (Fig 1Aiii). In these lesions, hCLEC-2-rFc2-binding sites predominantly appeared to co-localize with smooth muscle cells stained with the anti-smooth muscle actin antibody (Fig 1Ai; iv). In the DIT lesion, hCLEC-2-rFc2 binding appeared to have a membranous pattern (Fig 1Bi and ii). DIT lesions mostly consist of smooth muscle cells and very few macrophages (Fig 1Biii and iv) and hCLEC-2-rFc2-binding sites also co-localized with the smooth muscle cells (Fig 1Bi and iv).

### Recombinant CLEC-2, but not an anti-podoplanin antibody, bound to the intima of normal murine aortas

**Fig 2:**
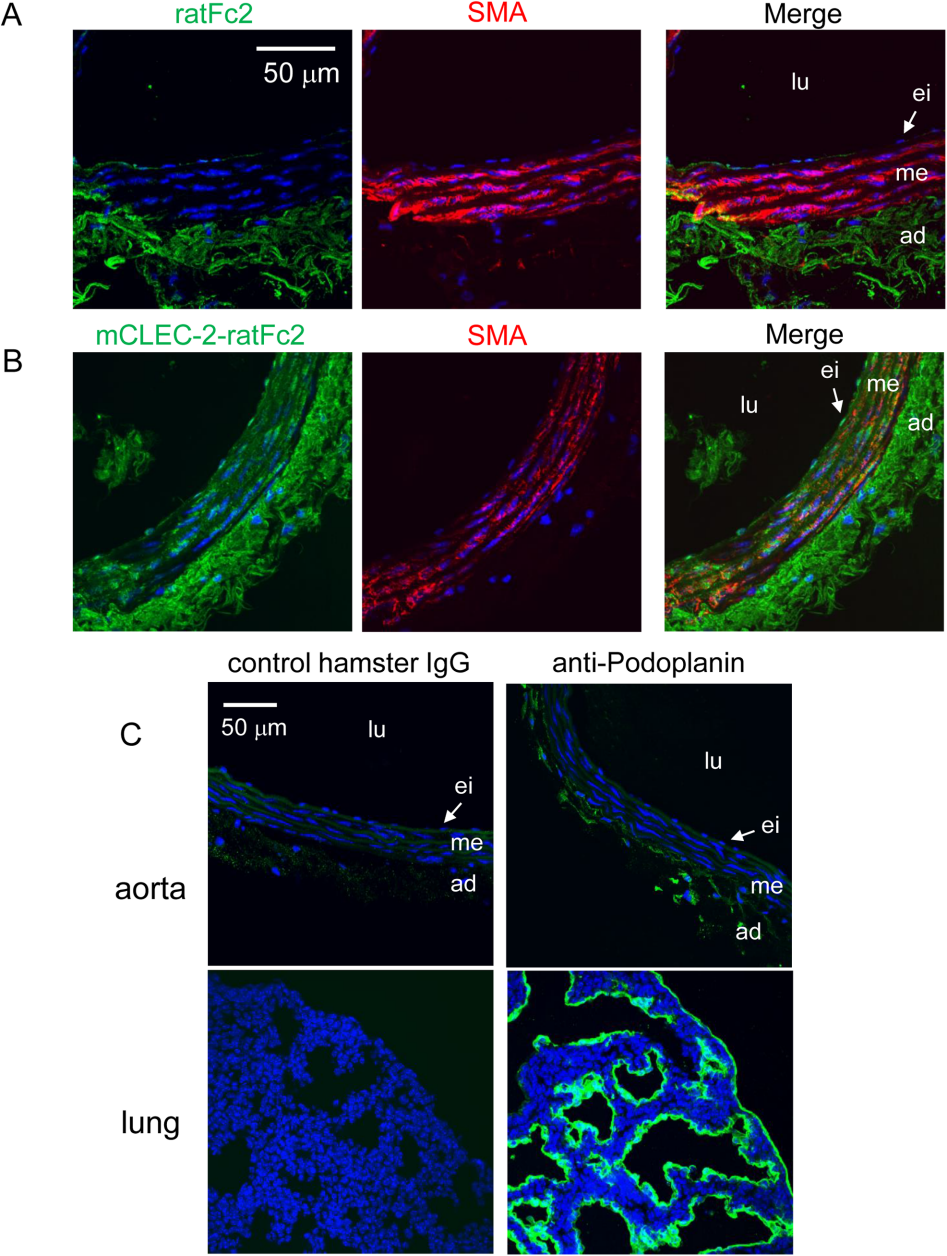
Recombinant CLEC-2, but not an anti-podoplanin antibody, bound to the normal mouse arterial wall. Frozen sections of the mouse abdominal aorta (A, B, C upper panels) or mouse lung (C, lower panels) were incubated with, control rat Fc2 (A, left panel), mCLEC-2-ratFc2 (B, right panel), anti-smooth muscle actin (SMA) antibody (A, B, middle panels), control hamster IgG (C, left panels), anti-mouse podoplanin antibody (C, right panels). The binding was visualized using anti-rat IgG Alexa Flour 488 (A, B, left panels), anti-goat IgG Alexa Flour 546 (A, B, right panels), or Alexa Fluor 488-labeled anti-hamster IgG (C). Left panels in A, B and middle panels in A, B were merged (A, B, right panels). Nuclei were counter-stained by DAPI (C). lu, lumen; ei, endothelial layer and intima; me, media; ad, adventitia.

Immunohistochemical analysis of the human aorta suggested that CLEC-2 ligands are expressed in VSMCs in early and advanced atheromatous lesions. These findings suggest that CLEC-2 ligands are present in both normal and atheromatous aortas. To confirm the presence of CLEC-2 ligands in the normal vessel wall, we immunohistochemically analyzed the normal murine aorta. We found that mouse CLEC-2-ratFc2, but not rat Fc2, bound to the frozen-thawed sections of the aortas obtained from WT C57Bl/6 mice, although non-specific binding of rat Fc2 was observed in adventitia (Fig 2A and 2B, left panels). This staining was co-localized with VSMCs of the intima stained with anti-smooth muscle actin antibody (Fig 2A and 2B, middle and right panels). Moreover, the anti-podoplanin antibody specifically bound to the normal murine lung tissue, but not to the normal murine aorta (Fig 2C), suggesting that the CLEC-2 ligands present in the intima of the normal arterial wall are not podoplanin.

### CLEC-2 ligands other than podoplanin are expressed on the surface of cultured coronary artery smooth muscle cells (CASMCs)

**Fig 3:**
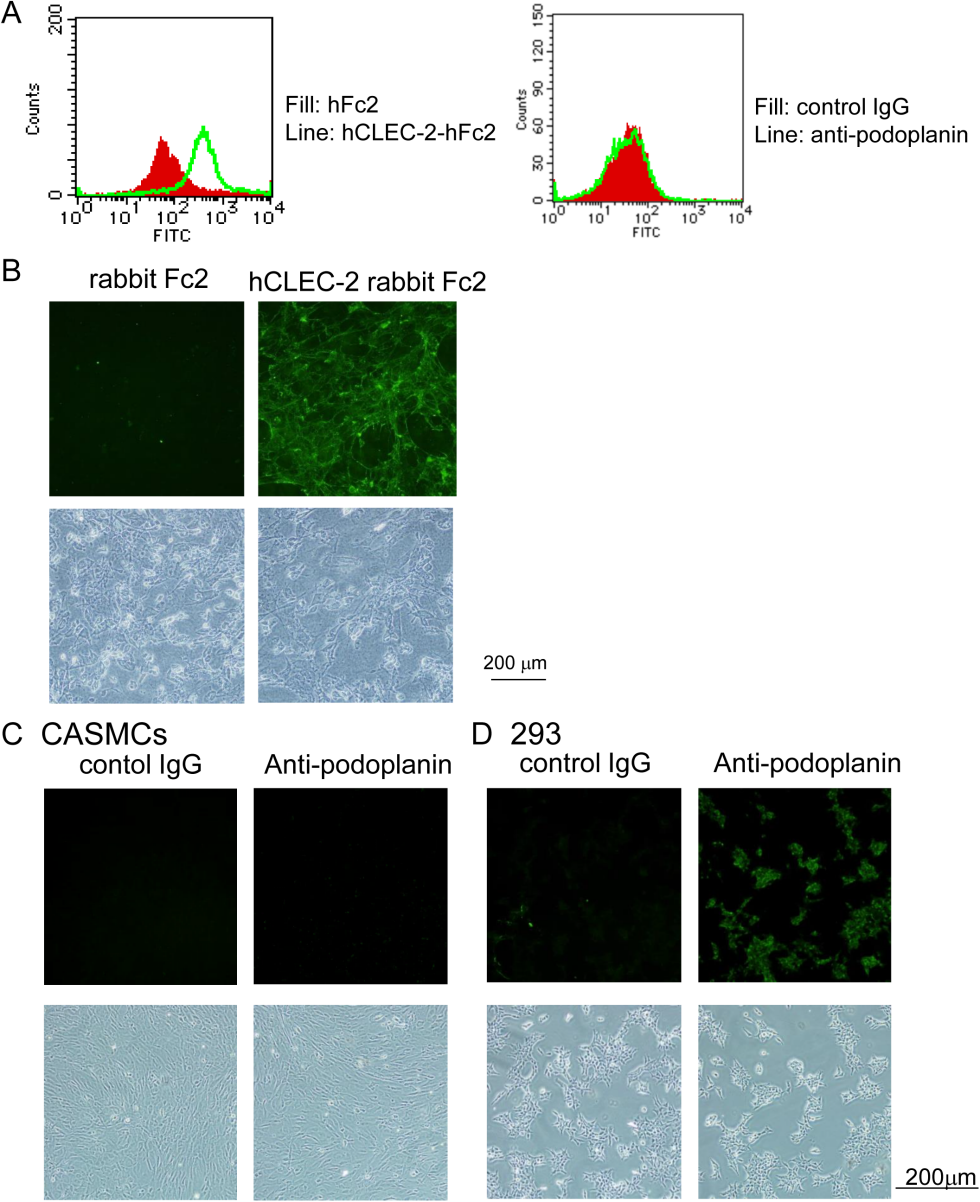
Recombinant CLEC-2, but not an anti-podoplanin antibody, bound to the surface of cultured coronary artery smooth muscle cells (CASMCs). A) CASMCs were incubated with hFc2, hCLEC-2-hFc2, control rat IgG, or anti-podoplanin antibody. After non-bound antibodies or proteins were removed, the cells were stained with anti-human IgG-FITC or anti-rat IgG-FITC, and analyzed using FACSCan. B-D) CASMCs (B, C) or 293TREx cells (293) (D) cultivated on a 24-well culture dish were incubated with rFc2, hCLEC-2-rFc2, control rat IgG, or anti-podoplanin antibody. After washing, cells were stained with anti-rabbit or anti-rat IgG labeled with Alexa Fluor 488. After washing, cells were visualized by fluorescence microscopy (upper panels) or phase-contrast Microscopy (lower panels).

To further investigate the hypothesis that CLEC-2 ligands are present in VSMCs, we analyzed CASMCs. Flow cytometric analysis demonstrated that hCLEC-2-rFc2 clearly bound to CASMCs (Fig 3A left panel), whereas podoplanin was not expressed in CASMCs (Fig 3A, right panel), suggesting that CASMCs express a ligand for CLEC-2 that is distinct from podoplanin. Furthermore, immunocytochemical analysis revealed the binding of hCLEC-2-rFc2, but not rFc2 or the anti-podoplanin antibody, to CASMCs (Fig 3B and 3C). In contrast, podoplanin-expressing 293TREx cells were clearly stained by the antibody (Fig 3D), further confirming that CASMCs express a ligand for CLEC-2 that is distinct from podoplanin.

### CASMCs induce platelet activation depending on the presence of CLEC-2 in platelets

**Fig 4:**
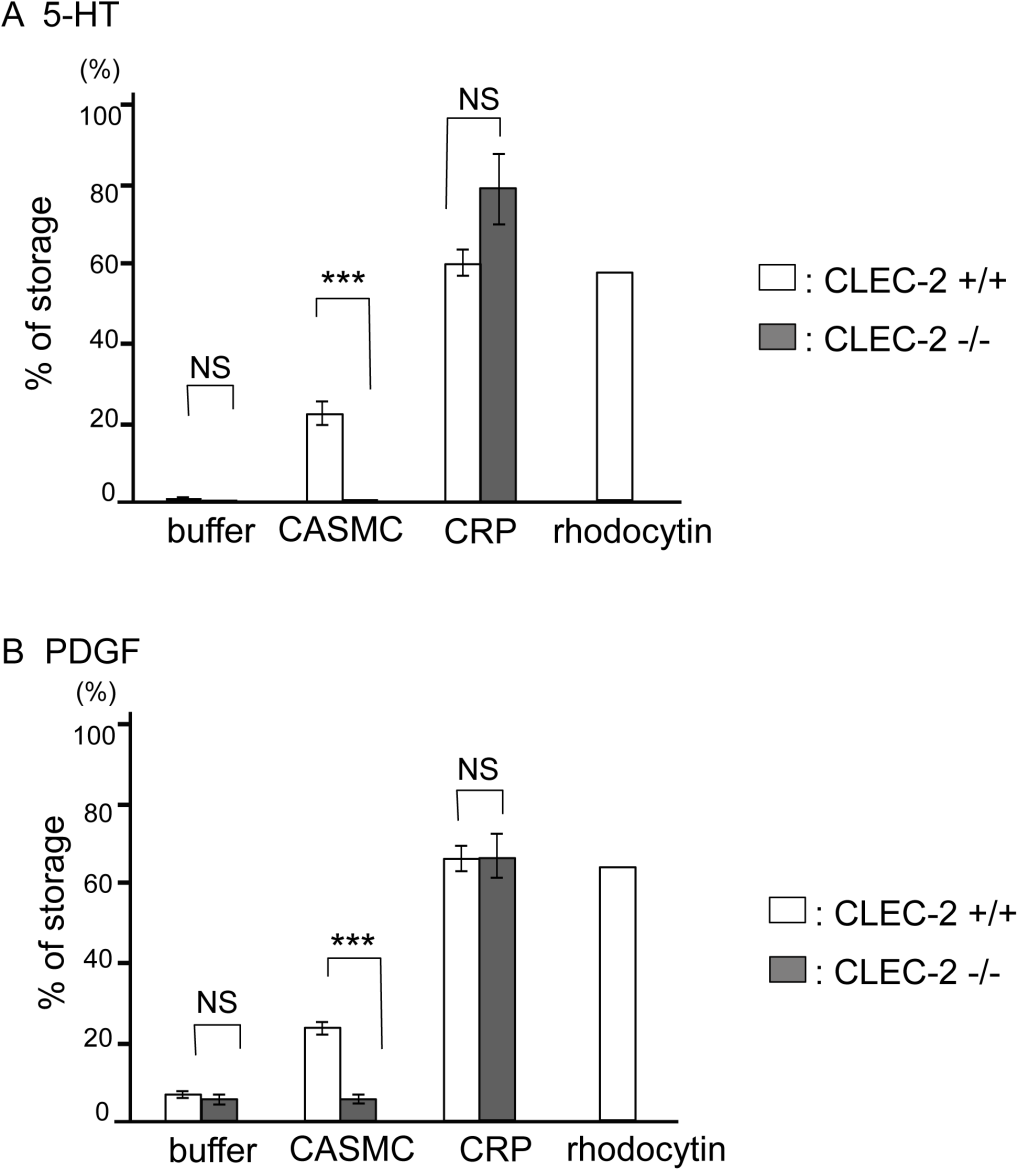
Coronary artery smooth muscle cells (CASMCs) stimulated release of α and dense granule contents through CLEC-2. Washed platelets obtained from the CLEC-2-deficient chimeras were incubated with buffer, CASMCs, CRP, rhodocytin, or lysis buffer for 10 min. The platelets were centrifuged. The amount of secreted 5-hydroxytryptamine (5-HT) (A) or platelet-derived growth factor (PDGF) (B) in the supernatants was measured by ELISA. Platelet lysates were used to measure the total amount of 5-HT or PDGF stored in platelets. The results were expressed as the percentage of secreted 5-HT or PDGF relative to the total amount stored in platelets ± SE (A. n = 9 from three independent experiments, B. n = 12 from four independent experiments). Three asterisks denote p < 0.005.

The finding that CASMCs express CLEC-2 ligands led us to investigate whether these ligands are able to activate platelets by binding to CLEC-2. To address this issue, we utilized chimeric mice that were lethally irradiated and rescued by fetal liver cell transplantation from CLEC-2^−/−^ embryos (CLEC-2-deficient chimeras) or CLEC-2^+/+^ embryos (wild-type (WT) chimeras). We had to resort to the use of CLEC-2 chimeric mice because CLEC-2^−/−^ mice are embryonic/neonatal lethal. The CLEC-2-deficient chimeras manipulated in this manner lack CLEC-2 expression in blood cells[6]. We prepared a mixture of washed murine platelets with suspended CASMCs and incubated them under stirring conditions. After the indicated period, the supernatant concentrations of 5-hydroxytryptamine (5-HT) and platelet-derived growth factor (PDGF), which are released upon platelet activation from dense and α granules, respectively, were determined. As shown in Fig 4, CASMCs stimulated the release of 5-HT and PDGF from WT platelets, but not from CLEC-2-deficient platelets, suggesting that CASMCs activate platelets depending on the presence of CLEC-2. A GPVI agonist, CRP, induced the release of 5-HT and PDGF in CLEC-2-deficient platelets in amounts similar to those observed in WT platelets, suggesting that the machinery for granule release is intact in CLEC-2-deficient platelets. Because CASMCs were washed using PBS prior to addition into washed platelets, it is suggested that CASMC membrane proteins or soluble factors released from CASMCs that adhere to CASMC surfaces stimulate platelets by binding to CLEC-2. The maximum concentration of rhodocytin, a potent CLEC-2 agonist, resulted in release of approximately 60% 5-HT or PDGF, whereas CASMCs stimulated 20% release from WT platelets. The discrepancy in granule release may be explained by the insufficient stimulation of platelets by the CLEC-2 ligands on the surfaces of large CASMCs; in contrast, soluble rhodocytin could interact efficiently with CLEC-2 on platelets. CASMCs in suspension did not stimulate platelet aggregation (S1 Fig), which could be explained using the same mechanism. Alternatively, weak stimulation of platelets by CASMCs may be related to the efficacy of the putative CLEC-2 ligands on CASMCs to activate platelets; this remains an issue to be addressed.

### CASMCs stimulate thrombus formation under flow conditions depending on the presence of CLEC-2 in platelets

**Fig 5:**
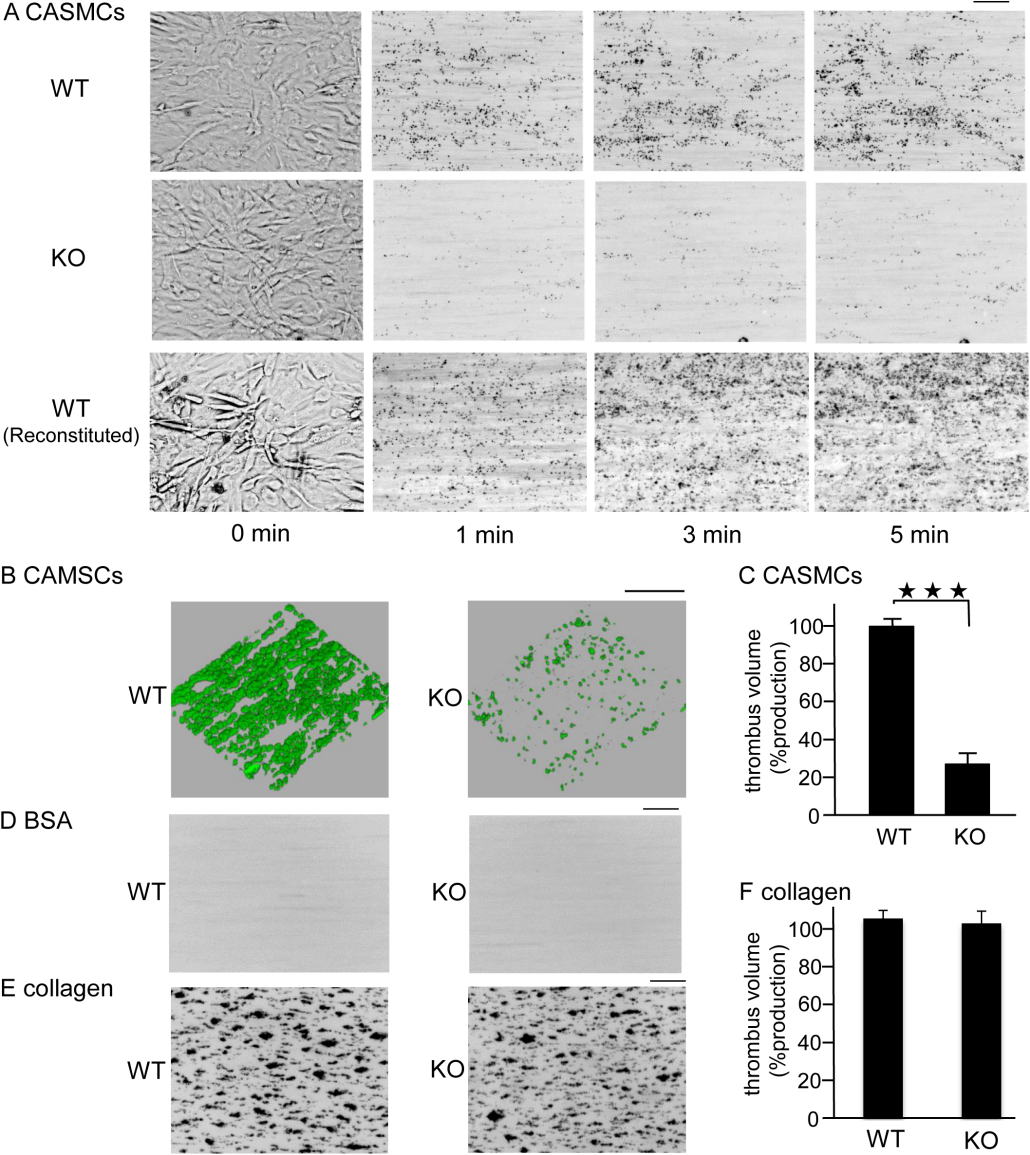
Coronary artery smooth muscle cells (CASMCs) induced thrombus formation under flow conditions through CLEC-2. A) GFP-positive CLEC-2-deficient whole blood (KO), GFP-positive wild type whole blood (WT), or wild type reconstituted blood (WT reconstituted) was perfused into cell microscopy chambers coated with CASMCs for 5 min at a shear rate of 400 s^−1^. Adherent platelets were visualized using a fluorescence video microscope with (1, 3, 5 min) or without (0 min) fluorescence. Movie data were converted into sequential photo images. B) After 5 min of perfusion, adherent platelets were visualized by confocal laser microscopy. C) The z-stack data of B were quantified. The thrombus volume was expressed as the cIFI per image (404374 03BCm^2^). The graph illustrates the percentage of the control (GFP-positive CLEC-2^+/+^ blood) cIFI ± SE (n = 6, from two independent experiments). Three asterisks denote p < 0.005. D, E) CLEC-2-deficient whole blood (KO) or wild type whole blood (WT) stained with DiOC_6_ was perfused into cell microscopy chambers coated with BSA (D) or collagen (E) for 5 min at a shear rate of 400 s^−1^. Adherent platelets at 5 min were visualized using a fluorescence video microscope with fluorescence. Movie data were converted into photo images. F) After 5 min of perfusion to collagen, adherent platelets were visualized by confocal laser microscopy, and the z-stack data were analyzed as described in C. Horizontal bars indicate 100 ∝m.

We investigated whether immobilized CASMCs induce thrombus formation under flow conditions, which presumably represent physiological conditions. Whole blood from WT chimeras anti-coagulated with heparin and PPACK was allowed to flow onto immobilized CASMCs at a shear rate of 400 s^−1^. We were not able to examine thrombus formation at higher shear rates because CASMCs detached from the surfaces of cell microscopy chambers (data not shown). Whole blood from WT chimeras formed large platelet aggregates, whereas that from the CLEC-2-deficient chimeras only showed sparse platelet adhesion without large platelet aggregates (Fig 5A). Reconstituted blood consisting of washed red blood cells and washed platelets also formed aggregates on the surface of CASMCs, suggesting that a direct association between CASMCs and platelets, rather than indirect association via plasma components, stimulates platelet activation. Quantification of the thrombus volume clearly indicated that thrombus formation was significantly impaired in CLEC-2-deficient whole blood (Fig 5B), suggesting that CASMCs activate platelets through CLEC-2 to induce thrombus formation. Whole blood from both types of mice did not form thrombi on the BSA-coated surfaces (Fig 5C).

We and others have previously reported that thrombus volume in collagen-coated capillaries under high shear flow conditions (1500–2000 s^-1^) is inhibited in the absence of CLEC-2, and proposed that the ligand(s) of CLEC-2 is present on the surface of activated platelets (we proposed homophilic association of CLEC-2)[6, 10]. CLEC-2 does not bind to collagen, but thrombus formation on collagen-coated surfaces was reduced by 20% in CLEC-2 deficient blood, suggesting that a CLEC-2 ligand is present on the surface of platelets[6]. Thus, the reduced thrombus formation of CLEC-2-deficient blood on the surface of CASMCs may be at least partially due to the lack of homophilic association of CLEC-2. However, under low shear (400 s^-1^), thrombus volume was not significantly inhibited on collagen-coated surfaces in CLEC-2-deficient blood (Fig 5E), whereas thrombus formation was reduced by 80% on CASMCs-coated surfaces in CLEC-2 deficient blood (Fig 5B). Moreover, CLEC-2 binding sites distinct from podoplanin were observed on the surface of CASMCs (Fig 3) and CASMCs stimulated 5HT release from platelets in a CLEC-2 dependent manner (Fig 4). These findings suggest that the decrease in thrombus formation of CLEC-2-deficient blood on the surface of CASMCs is not only due to the lack of homophilic association, but also to the lack of association between CLEC-2 and its ligand on CASMCs.

### *In vivo* thrombus formation induced by ferric chloride (FeCl_3_), but not by photochemical injury, is impaired in CLEC-2-deficient chimeras

Exposure of VSMCs to blood flow can occur upon plaque erosion or rupture in atherosclerotic arteries. To investigate the pathophysiological relevance of the binding between CLEC-2 and its ligands on the surface of VSMCs, we used two types of *in vivo* thrombosis models: the FeCl_3_ model and the PIT model. In the FeCl_3_ model, the femoral artery of the anesthetized mice is injured by topical application of FeCl_3_, which causes endothelial damage[22]. In the PIT model, thrombus formation is triggered by damage to the endothelium of the femoral artery irradiated by green light after intravenous injection of rose Bengal. It is believed that the endothelial damage is caused by reactive oxygen species generated at the site of rose Bengal excitation. In both cases, the time required for vessel occlusion by thrombus formation can be evaluated using a laser Doppler velocimeter.

**Fig 6:**
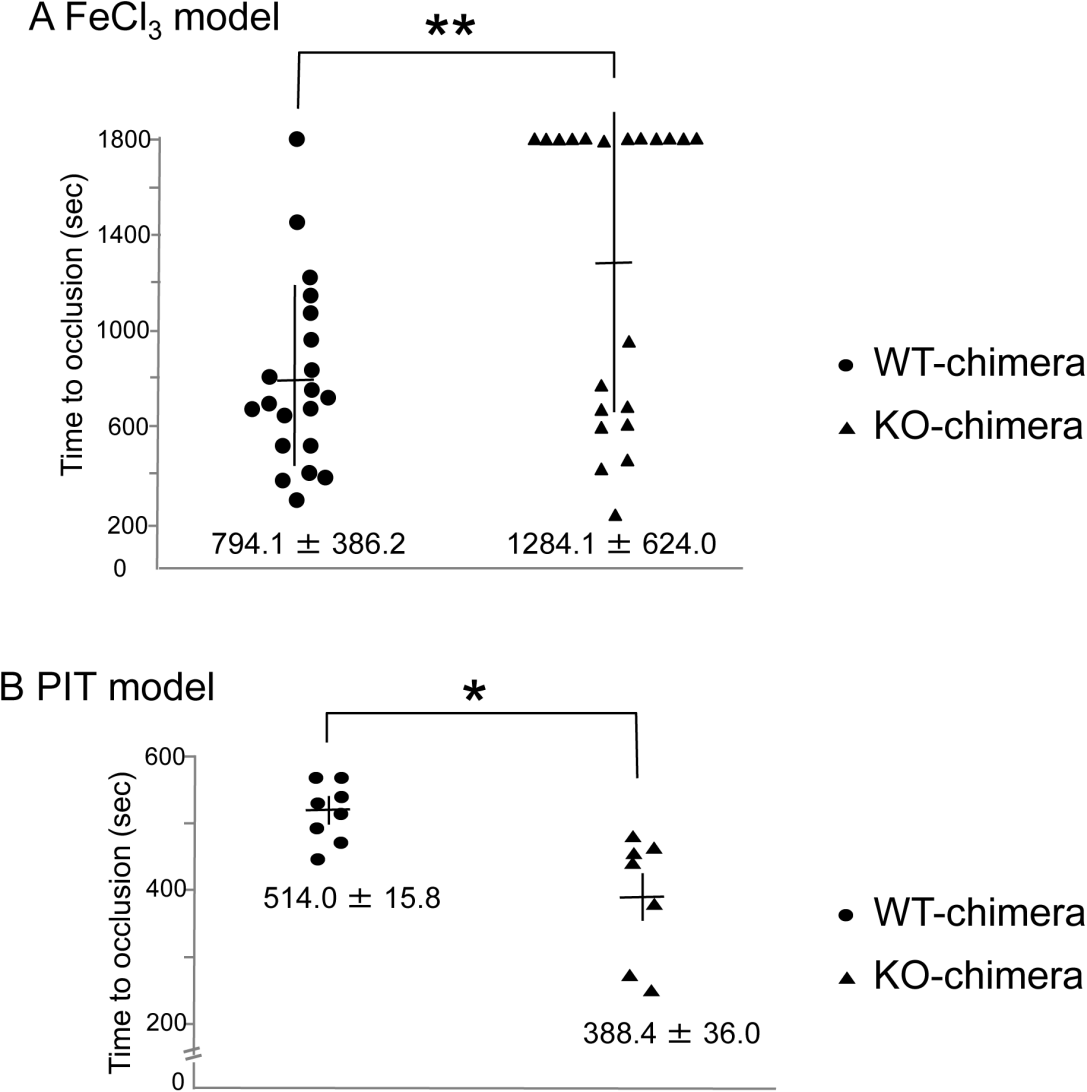
Time to thrombotic occlusion of vessels stimulated by FeCl_3_, but not by green light and dye, was significantly delayed in the CLEC-2-deficient chimeras. The femoral artery of anesthetized wild type (WT) chimeras or CLEC-2-deficient chimeras was exposed by blunt dissection. A) Time to vessel occlusion in the FeCl_3_ model: a 1strip of filter paper saturated with 10% FeCl_3_ was applied to the adventitial surface of the exposed femoral artery. B) Time to occlusion in the photochemically induced thrombosis (PIT) model. After 5 min of irradiation to the exposed femoral artery, rose Bengal was infused. The time to thrombotic vessel occlusion of the artery was measured by monitoring femoral blood flow using a Doppler blood flow velocimeter. Each symbol represents one individual. The mean time to occlusion ± SE is indicated in the graphs. Two asterisks denote p < 0.01.

In the FeCl_3_ model, the time to occlusion was significantly prolonged in CLEC-2-deficient chimeras compared with WT chimeras (Fig 6A). CLEC-2 chimeras showed only marginal reduction in platelet counts (CLEC-2-deficient chimeras, 92.42/μL ± 7.913/μL vs. WT chimeras, 94.53/μL ± 8.978/μL), but the difference was not significant. Furthermore, since only severe thrombocytopenia is required for defective thrombus formation in mice[23], this slightly reduced platelet count cannot explain the cause of longer occlusion time in CLEC-2-deficient chimeras. In contrast, in the PIT model, the time to occlusion was shorter in CLEC-2-deficient chimeras than in WT-chimeras (Fig 6B). In the PIT model, no difference was observed in platelet counts between WT chimeras (87.8/μL ± 15.7/μL) and CLEC-2 chimeras (92.7/μL ± 3.45/μL). We found that in the PIT model, the internal elastic lamina, which separates the tunica intima from the smooth muscle cell-rich tunica media, is intact (compare S2A and S2B Fig), as previously reported[24]. However, we found that the internal elastic lamina was lacerated in the FeCl_3_ model (compare S2A and S2C Fig), which suggests that VSMCs are exposed to blood flow. These findings suggest that the exposed smooth muscle cells in the FeCl_3_ model contributed to thrombus formation through binding of CLEC-2 with a putative CLEC-2 ligand on the surface of smooth muscle cells. The occlusion time was not prolonged in CLEC-2-deficient chimeras compared with WT chimeras in the PIT model. These findings could be attributed to the lack of contact between smooth muscle cells and platelet CLEC-2 in the blood.

### S100A13 is a possible CLEC-2 ligand in CASMCs

**Fig 7:**
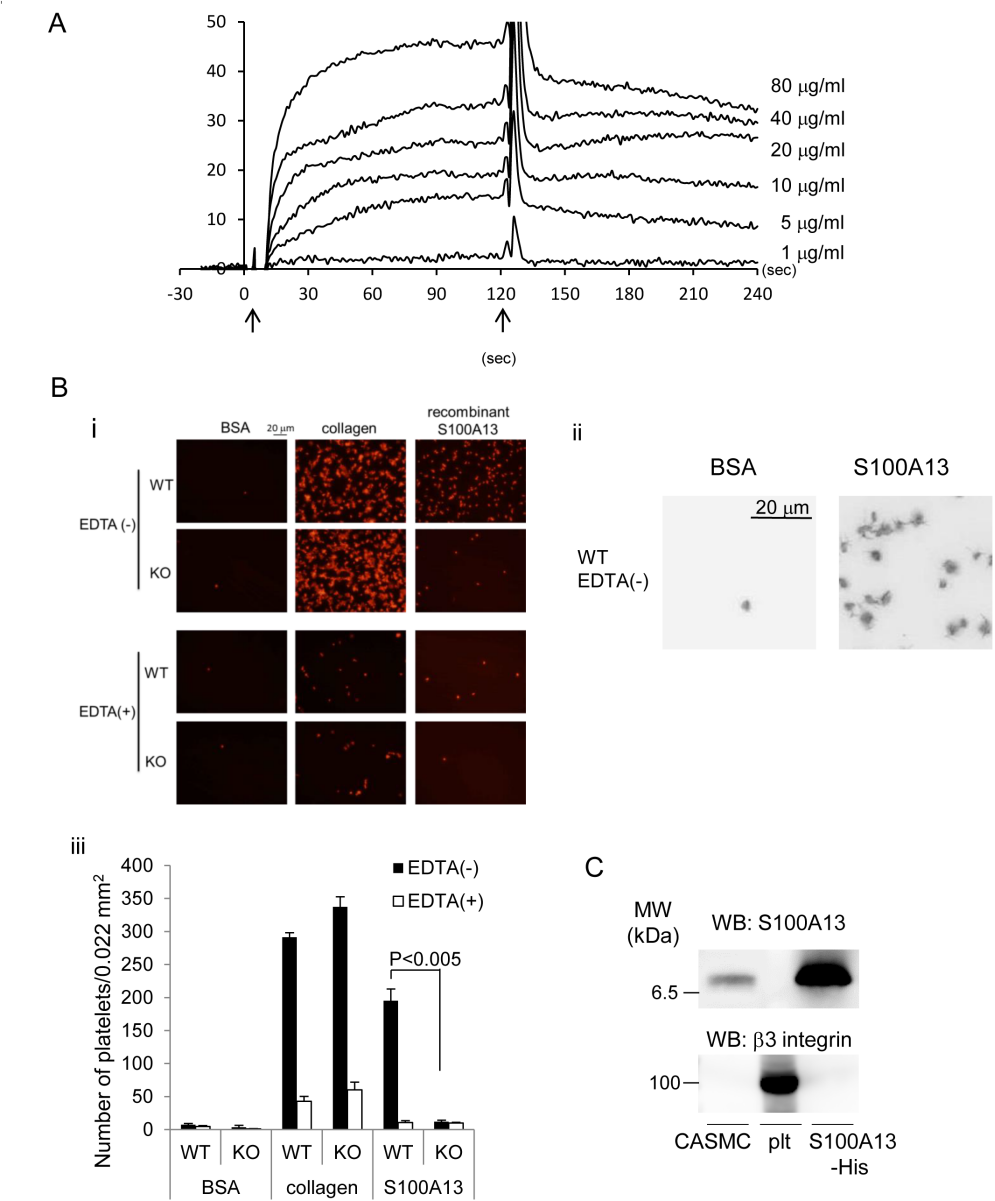
Recombinant S100A13 protein associated with CLEC-2. A) Different concentrations of His-S100A13 were flowed over an immobilized hCLEC-2-rFc2 or a control rFc2-coated surface. The arrows indicate the beginning and the end of perfusion of S100A13. The results from one experiment are shown that is representative of the other three. RU indicates resonance units. B) (i) Platelets spreading on the surface of BSA, collagen, or S100A13 were investigated. (ii) Magnified images of adhered WT platelets on the surfaces coated with BSA or recombinant S100A13. (iii) Quantification of adherent platelets in the images in (i). Adherent platelets were counted. C) Western blotting with anti-S100A13 or anti-β3 integrin antibody. Plt represents platelets. The data are representative of three experiments.

We attempted to identify CLEC-2 ligands on the surface of CASMCs. Because we failed to identify the ligands by MS/MS analysis of CASMC proteins associated with recombinant CLEC-2, we next performed a protein array using a ProtoArray® Human Protein Microarray to identify potential interactions. The ProtoArray® has 9400 proteins spotted on the surface of a nitrocellulose-coated glass slide. We detected proteins on the array associated with biotin-labeled hCLEC-2-hFc2, but not with biotin-labeled hFc2 using avidin-HRP. We did not find positive binding of membrane proteins. Therefore, we focused on three secreted proteins reportedly expressed in CASMCs and verified the interaction between recombinant CLEC-2 and these proteins using surface plasmon resonance. Biacore analysis showed that recombinant S100A13-GST, but not the other two proteins (data not shown), associated with the recombinant CLEC-2 (Fig 7A) with a binding Kd of 1.37 × 10^−7^ M. An association between His-tagged recombinant S100A13 and hCLEC-2-rFc2 was also observed (data not shown), suggesting that the association was not via the tagged proteins, but via S100A13. S100A13 belongs to the S100 protein family, which is a part of the Ca^2+^-binding protein superfamily with an EF hand domain. S100A13 is an intracellular protein, which plays a crucial role in the non-classical pathway for extracellular release of FGF-1 and IL-1α, both of which lack signal peptides, by forming heterotetrameric complexes [25–27].

We utilized CLEC-2-deficient platelets to confirm the association between native CLEC-2 on the surface of platelets and S100A13. As shown in Fig 7B (i and ii), CLEC-2-deficient platelets adhered to collagen-coated surfaces to the same extent as WT platelets. In contrast, platelet adhesion to the surface of recombinant S100A13 was greatly and significantly inhibited with CLEC-2-deficient platelets. In the presence of EDTA, even adhesion of WT platelets to S100A13-coated surfaces was markedly inhibited. We found that exogenous addition of S100A13 did not induce platelet aggregation or 5-HT/PDGF release from suspended murine platelets (data not shown). However, a magnified image of adhered platelets on a surface coated with S100A13 clearly showed platelet spreading (Fig 7Biii, right panel). In contrast, platelets nonspecifically adhered to a surface coated with BSA showed no morphological changes (Fig 7Biii, left panel). These findings clearly demonstrate that CLEC-2 associates with S100A13 dependent on divalent cations and that interaction between CLEC-2 and S100A13 results in platelet activation. Western blotting revealed that S100A13 was expressed in CASMCs, but not in platelets (Fig 7C, upper panel). A positive control staining of anti-β3 integrin antibody showed presence of platelet protein in the middle lane of Fig 7C upper panel. Expression of S100A13 in CASMCs is consistent with a previous report [28].

### S100A13 is expressed in the luminal area of atherosclerotic lesions

**Fig 8:**
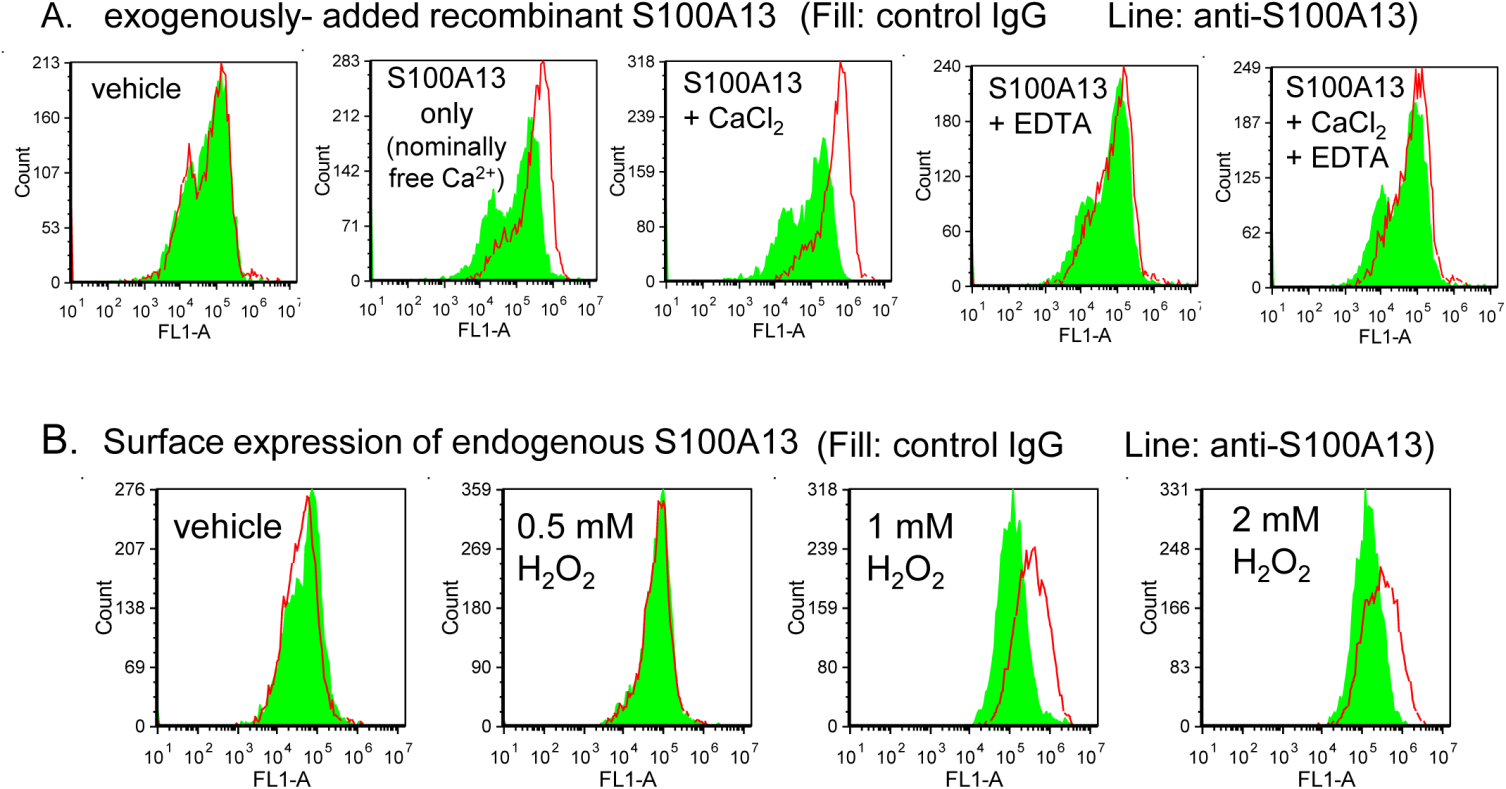
S100A13 is not expressed on the surface of coronary artery smooth muscle cells (CASMCs), but oxidative stress induced surface expression of S100A13. A) Binding of recombinant S100A13 on the surface of CASMCs were examined by flow cytometory. CASMCs were preincubated with vehicle only (1st panel) or recombinant S100A13 in the presence of vehicle (2^nd^ panel), 0.1 mM CaCl_2_ (3^rd^ panel), 1 mM EDTA (4^th^ panel), or 0.1 mM CaCl_2_ + 1 mM EDTA (5^th^ panel). After excess of the recombinant protein was removed by centrifugation, cells were incubated with control mouse IgG (filled) or anti-S100A13 antibody (line), followed by Alexa Flour 488-conjugated anti-mouse IgG. B) Surface expression of endogenous S100A13 was analyzed by flow cytometry. CASMCs pretreated with indicated concentrations of H_2_O_2_ were incubated with control mouse IgG (filled) or anti-S100A13 antibody (line), followed by Alexa Flour 488-conjugated anti-mouse IgG.

Unfortunately, surface expression was not observed by flow cytometric analysis of unpermeabilized CASMCs (Fig 8A, left panel), clearly indicating that S100A13 is not responsible for 5HT/PDGF secretion induced by cultured CASMCs (Fig 4). Similar to suspended CASMCs, surface expression of S100A13 was not observed in adherent CASMCs, although S100A13 was detected in the cytoplasm of permeabilized CASMCs (S3 Fig). These findings also indicate that S100A13 is not responsible for thrombus formation under flow (Fig 5) and that another CLEC-2 ligand is also expressed on the surface of CASMCs. Although S100A13 was not expressed on the surface of cultured CASMCs (Fig 8A, 1^st^ panel) exogenously added S100A13 recombinant protein was able to bind to the surface of CASMCs depending on divalent cations (Fig 8B, compare 2^nd^ to 5^th^ panels).

It has been reported that S100A13 is released from the cytoplasm in response to stress, including heat shock[29] and serum depletion[30]. We next examined whether oxidative stress by H_2_O_2_ stimulated S100A13 release from CASMCs, because oxidative stress has been implicated in the pathogenesis of vascular abnormalities such as atherosclerosis and hypertension[31]. As shown in Fig 8B, pretreatment with H_2_O_2_ dose-dependently stimulated surface expression of S100A13. Thus, it appears that S100A13 binds to the surface of CASMCs upon their release from the cytoplasm and could be a stimulatory ligand for CLEC-2 in certain physiological/pathological settings *in vivo*.

**Fig 9:**
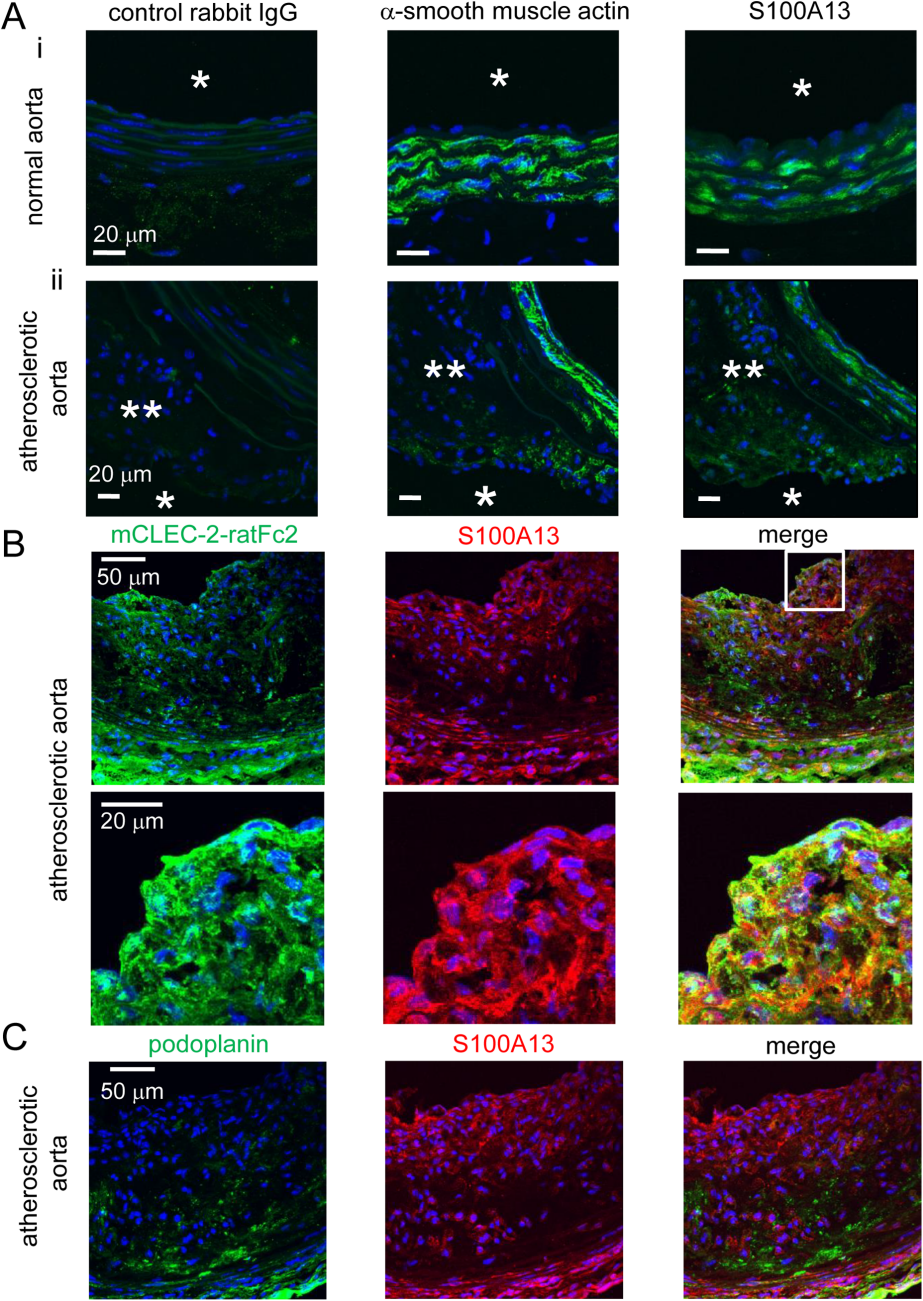
S100A13, but not podoplanin, was expressed on the surface of atherosclerotic lesions. A) Serial frozen-thawed sections of the mouse abdominal aorta from wild type (i) or ApoE- deficient mice fed with high fat diet (ii) were incubated with control rabbit IgG (left panel), anti-smooth muscle actin (middle panel), anti-S100A13 antibody (right panel), followed by anti-rabbit IgG-Alexa Flour 488. Single and double asterisks indicate lumen and atherosclerotic plaque, respectively. B) Serial frozen-thawed sections of the mouse abdominal aorta from ApoE- deficient mice fed with high fat diet were incubated with mCLEC-2-ratFc2 (left panel), anti-S100A13 antibody (middle panel), followed by anti-rat IgG-Alexa Flour 488 or anti-rabbit IgG Alexa Flour 546. Images in left and middle panels were merged (right panel). Lower panels are magnified images of the squared area in the upper panels. C) Serial frozen-thawed sections of the mouse abdominal aorta from ApoE- deficient mice fed with high fat diet were incubated with anti-podoplanin antibody (left panel), anti-S100A13 antibody (middle panel), followed by anti-hamster IgG-Alexa Flour 488 or anti-rabbit IgG Alexa Flour 546. Images in left and middle panels were merged (right panel). All the sections were counter-stained with DAPI.

To investigate the surface expression of S100A13 under pathological conditions *in vivo*, we histologically analyzed whether S100A13 is expressed in the normal aorta of WT mice and the atherosclerotic aorta of ApoE-deficient mice fed a high fat diet. As shown in Fig 9Ai, in the normal aorta, positive staining of S100A13 (right panel) was observed in the SMC-positive area (middle panel), but not in the luminal area (right panel, arrow), which can be exposed to the blood stream. In the atherosclerotic aorta (Fig 9Aii), positive staining of S100A13 (right panel) was observed in the SMC-positive area (middle panel), similar to the normal aorta. However, the S100A13-positive area was observed in the luminal area (right panel, arrow), distinct from the normal aorta. Moreover, co-staining for S100A13 and SMC showed the specific expression of S100A13 in the luminal area of atherosclerotic aorta (S4 Fig, Fig 9B and 9C middle panels). Fig 9B showed that most of the mCLEC-2-ratFc2 binding sites were identical to S100A13 antibody binding sites. These findings suggest that S100A13, but not podoplanin, is expressed on the surface of the vessel wall and can interact with CLEC-2 in atherosclerotic lesions.

We found that oxidative stress did not induce podoplanin expression on the surface of CASMCs (S5 Fig). Podoplanin was not expressed in the normal vessel wall (Fig 2C). In the case of atherosclerotic aorta, however, podoplanin was expressed inside of the plaque, but not expressed on the surface of plaque (Fig 9C). On the other hand, S100A13 was expressed in the luminal area of atherosclerotic aorta (Fig 9C). Based on these findings, we suggest that S100A13 and an unknown ligand on the surface of CASMCs, but not podoplanin, contribute to thrombus formation without plaque rupture.

**Fig 10:**
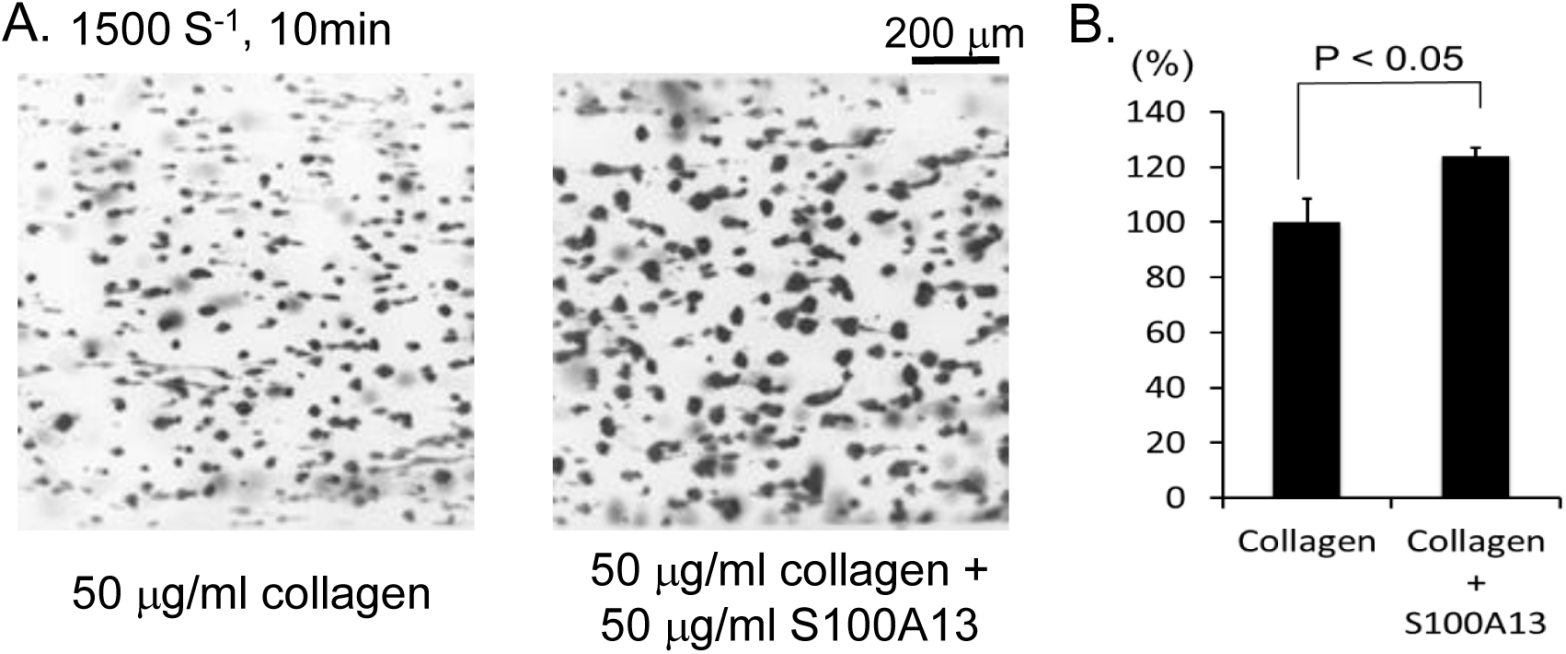
S100A13 potentiated thrombus formation on collagen-coated surfaces under flow. (A) Anti-coagulated human whole blood was perfused into cell microscopy chambers coated with collagen or collagen plus S100A13 for 10 min at a shear rate of 1500 s^−1^. Adherent platelets were visualized using a fluorescence video microscope. Movie data were converted into sequential photo images. (B) Thrombus volume was quantified. After 5 min of perfusion, adherent platelets were visualized by confocal laser microscopy, and the z-stack data were analyzed. The thrombus volume was expressed as the cIFI per image (404374 μm^2^). The graph illustrates the percentage of the surfaces coated with collagen only cIFI ± SE (n = 6, from two independent experiments).

We next investigated whether immobilized S100A13 stimulates thrombus formation under flow conditions. Thrombus formation was not observed in a glass capillary coated with S100A13 under flow (data not shown). However, thrombus formation at an arterial shear (1500 S^-1^), but not that at venous shear (400 S^-1^, data not shown), was significantly greater in the capillary coated with collagen and S100A13 than in the capillary coated with collagen only (Fig 10). To investigate whether the increased thrombus formation was dependent on CLEC-2, whole blood from WT or CLEC-2 chimeras anti-coagulated with heparin and PPACK was allowed to flow onto the surfaces coated with collagen only or collagen plus S100A13 at a shear rate of 1500 s^−1^. In the case of WT chimeras, thrombus formation on the surface of collagen plus S100A13 tended to be increased compared with that on the surface of collagen only, although there was no statistical significance (S7 Fig). In the case of CLEC-2-chimeras, however, thrombus formation was identical to that in collagen plus S100A13. As others and we reported previously[6, 10], thrombus volume in collagen-coated capillaries under high shear flow conditions (1500 s^-1^) was significantly inhibited in the absence of CLEC-2 (S7 Fig). These findings suggest that S100A13 potentiates thrombus formation on surfaces coated with collagen under flow through CLEC-2 and that S100A13 and another CLEC-2 ligand on the surface of VSMCs may contribute to thrombus formation at the site of atherosclerotic lesions *in vivo*, although *in vitro* platelet activation by CASMCs cannot be explained by S100A13 alone.

## Discussion

In the present study, we propose that CLEC-2 ligands other than podoplanin are expressed on the surface of both normal and pathologic VSMCs and contribute to thrombus formation through CLEC-2 *in vivo* and *in vitro*.

We found that recombinant CLEC-2 bound to advanced atherosclerotic lesions (fibro fatty plaques). Hatakeyama et al. reported that podoplanin, an endogenous ligand of CLEC-2, is expressed in advanced atherosclerotic lesions of the human aorta, implying that podoplanin is the counterpart of CLEC-2 in advanced lesions[32]. However, they also showed that podoplanin is essentially not expressed in early atherosclerotic lesions (DIT), the intima of which is primarily composed of smooth muscle cells. We found extensive binding of hCLEC-2-rFc2 in early lesions, and binding sites co-localized with VSMCs. Therefore, it is clear that CLEC-2 ligands other than podoplanin exist in the arterial wall and are particularly associated with VSMCs, although podoplanin may also act as a CLEC-2 ligand in advanced atherosclerotic lesions. Moreover, podoplanin was found inside of the advanced atherosclerotic lesions, but not on the surface of the lesions in human [32] and mice (Fig 9C). These findings suggest that podoplanin cannot have access to CLEC-2 unless the plaque ruptures and that S100A13, but not podoplanin, is expressed on the surface of atherosclerotic lesions and can interact with CLEC-2 in the blood flow.

Since primary VSMCs rapidly dedifferentiate under culture conditions[33], we sought to determine whether hCLEC-2-rFc2 binds to “normal” VSMCs. Because we could not obtain normal human aorta samples, we used mouse aortas. Histological analysis showed binding of mouse CLEC-2-rat Fc2 in the SMA-positive area of the wall of normal aortas (Fig 2), confirming that CLEC-2 ligands other than podoplanin are present in the wall of normal arteries. Moreover, flow cytometric analysis showed that hCLEC-2-rFc2, but not anti-podoplanin antibody, bound to non-permeabilized cultured CASMCs (Fig 3). On the basis of these findings, the existence of CLEC-2 ligands other than podoplanin on the surface of CASMCs is confirmed.

Suspended CASMCs stimulated the release of α granule and dense granule contents from platelets in a CLEC-2-dependent manner (Fig 4), but did not induce platelet aggregation (S1 Fig). Binding between CLEC-2 and the ligand on CASMCs induced thrombus formation under flow conditions (Fig 5B), which represents a more physiological condition than the static conditions under which the platelet aggregation experiment was performed. We assume that the decrease in thrombus formation of CLEC-2-deficient blood on the surface of CASMCs is mainly due to the lack of association between CLEC-2 and its ligand on CASMCs, and that the lack of CLEC-2 homophilic association is only minimally, if at all, involved, based on the following findings: 1) Thrombus formation on collagen-coated surfaces, which do not have a CLEC-2 binding site, was not inhibited in CLEC-2-deficient platelets under low shear (Fig 5E and 5F); 2) CASMCs clearly expressed a CLEC-2 ligand (Fig 2); 3) CASMCs stimulated α and dense granule release from platelets in a CLEC-2-dependent manner and released secondary mediators that play a crucial role in thrombus formation under flow[34]. It is strongly suggested that a CLEC-2 ligand on the surface of CASMCs contributes to CLEC-2-dependent thrombus formation, at least under low shear conditions.

We observed that the internal elastic lamina of the murine femoral artery was lacerated in an *in vivo* thrombosis model induced by FeCl_3_ (S2 Fig) and that hCLEC-2-rFc2 bound to frozen-thawed sections of the normal murine artery (Fig 2). The time to occlusion in the FeCl_3_-injured artery was significantly prolonged in CLEC-2-deficient chimeras (Fig 5), suggesting that normal VSMCs are exposed to blood flow in this FeCl_3_ model and that normal VSMCs also stimulate thrombus formation through binding between CLEC-2 and its ligands (unknown ligand on the surface of VASCs and/or S100A13). Co-staining of S100A13 and smooth muscle actin showed that expression of S100A13 in VSMCs of the artery injured by FeCl_3_ (S6 Fig). Podoplanin is not involved in FeCl_3_-induced thrombus formation because podoplanin is not expressed in the normal vessel wall (Fig 2B) and the anti-podoplanin blocking antibody 8F11 did not prolong the time to occlusion in the FeCl_3_-injured artery (data not shown).

In contrast to the FeCl_3_ model, the time to occlusion in the photo-injured artery in the PIT model was not prolonged with WT chimeras, possibly because the internal elastic lamina is intact in the PIT model[24] and the CLEC-2 ligands in VSMCs are not exposed to blood flow (S2 Fig). In the PIT model, the time to occlusion of the CLEC-2-deficient chimera was slightly but significantly shortened compared with that of the WT chimera. We found that PT and APTT were not prolonged in CLEC-2-deficient chimeras (data not shown). The physiological significance and the mechanism of the shorter occlusion time in the CLEC-2-deficient chimera remain unclear.

We proposed that S100A13 is the CLEC-2 ligand based on protein microarray findings, surface plasmon resonance, and an adhesion assay in CLEC-2-deficient platelets (Fig 7). S100A13 is a Ca^2+^-binding protein that belongs to the S100 protein family. S100A13 crucially forms heterotetrameric complexes via non-classical pathways for the secretion of FGF-1 and IL-1α, both of which lack a signal peptide for classical secretion[25–27]. S100A13 has been implicated in angiogenesis[35–37], tumor development[38], and chronic inflammation[39]. S100A13 is released by stress, including heat shock[29] and serum depletion[30]. The receptor for advanced glycation end products (RAGE) has been shown to be a receptor for S100A13. We did not observe surface expression of S100A13 in washed and suspended CASMCs (Fig 8A) or in adherent CASMCs (S3 Fig). It is noteworthy that the same washed and suspended CASMCs (Fig 3A) or adherent CASMCs (Fig 3B) were obviously associated with hCLEC-2-rFc2 and stimulated granule release from platelets or thrombus formation under flow. These findings suggest that although we identified S100A13 as the CLEC-2 ligand in VSMCs, this protein is not responsible for stimulating granule release from platelets or thrombus formation under flow and that CLEC-2 ligands other than S100A13 are present. However, exogenously added S100A13 did bind to the CASMC surfaces (Fig 8A). In addition, oxidative stress induced surface expression of S100A13 (Fig 8B). Moreover, we observed that S100A13 was expressed in the luminal area and can be exposed to blood flow in atherosclerotic lesions (Fig 9) and that immobilized S100A13 potentiated thrombus formation on collagen-coated surfaces (Fig 10). Based on these findings, we suggest that S100A13 is released from CASMCs and bound to their surface under some pathological conditions, which may result in interactions between platelets and CASMCs *in vivo*. It has been reported that prominent staining of MAC-1, IL-1α, and S100A13 was observed at the site of neointimal thickening after balloon injury and that Cu^2+^ chelation inhibited formation of S100A13 heterotetrameric complexes and IL-1α release, thereby reducing neointimal thickening[40]. Therefore, S100A13 may be released and adhered to the surface of VSMCs, and stimulate platelets as the CLEC-2 ligand on the surface of VSMCs at the site of atherosclerotic lesions and stent insertion.

What is the pathophysiological significance of the CLEC-2-VSMC association? We suggest that this association plays a role in thrombosis at the site of stent implantation and plaque erosion, where VSMCs are exposed to blood flow. Upon stent implantation, VSMCs are exposed to blood flow before endothelialization occurs, particularly in the case of drug-eluting stents (DES), for which late stent thrombosis remains a significant problem. Recent findings utilizing intravascular ultrasound and optical coherence tomography suggested that delayed healing and neoatherosclerosis are important mechanisms for very late stent thrombosis[41]. The binding of CLEC-2 to a ligand in VSMCs may trigger stent thrombosis. Patients who undergo DES implantation have an almost lifelong risk of stent thrombosis and need to take dual anti-platelet therapy over an extended period because DES delays endothelialization. However, we have previously reported that CLEC-2-deficient bone marrow chimeric mice did not show a significantly increased bleeding tendency. Therefore, we speculate that an anti-CLEC-2 agent that inhibits the association between CLEC-2 and VSMCs has beneficial effects in preventing stent thrombosis by reducing the quantity of anti-platelet drugs required without increasing the frequency of bleeding complications. Plaque erosion likely occurs at smooth muscle cell/proteoglycan-rich plaques without a large lipid core, and is found in sudden death from coronary thrombosis, comprising 44% of all cases[42]. The association between CLEC-2 and its ligands in VSMCs may also be involved in this process. Blocking of the binding between CLEC-2 and VSMCs may be effective to prevent plaque erosion, which is more often observed in younger individuals and women.

## Supporting Information

S1 Fig CHO cells transfected with human podoplanin, but not CASMCs, induced platelet aggregation. Platelet aggregation by CHO cells transfected with human podoplanin (upper panels) or CASMCs (lower panels) was monitored by light transmission. Final concentrations of the cells were 2.5 × 10^5^ cells/ml (left panels) and 1.0 × 10^6^ cells/ml (right panels).
(TIF)

S2 Fig Internal media lamina was lacerated in FeCl_3_-injured femoral arteries, but not in photochemically-injured femoral arteries. A) Victoria blue-HE staining of non-injured murine femoral artery. Arrowheads indicates internal media lamina. B) Victoria blue-HE staining of photochemically-injured murine femoral artery. C) Victoria blue-HE staining of FeCl_3_-injured murine femoral artery. Laceration of internal media lamina is indicated by an arrow. The asterisks indicate thrombi.
(TIF)

S3 Fig S100A13 was expressed in the cytoplasm of adherent CASMCs, but not on the surface. CASMCs cultivated on a 24-well culture dish were fixed and incubated with control mouse IgG (upper panels) or anti-S100A13 antibody (63Y) (middle and lower panels), followed by anti-mouse IgG labeled with Alexa Fluor 488. Where indicated, cells were treated with 0.1% Triton X-100 before addition of the antibodies (lower panels). Cells were visualized by phase-contrast Microscopy (left panels) or fluorescence microscopy (right panels).
(TIF)

S4 Fig Co-staining for S100A13 and smooth muscle actin in the atherosclerotic lesion in mice. Frozen-thawed sections of the mouse abdominal aorta from ApoE-deficient mice fed a high fat diet were incubated with goat anti-α smooth muscle actin (left panels) or rabbit anti-S100A13 antibody (middle panels), followed by anti-rabbit IgG-Alexa Fluor 488 and anti-goat IgG-Alexa Fluor 597, respectively. Right panels are merged images of left and middle panels. Lower panels are magnified images of the square area from upper panels.
(TIF)

S5 Fig Oxidative stress induced surface expression of S100A13, but not that of podoplanin in CASMCs. Surface expression of endogenous S100A13 or podoplanin was analyzed by flow cytometry. CASMCs pretreated with vehicle (upper panels) or 1 mM H_2_O_2_ (lower panels) were incubated with control mouse IgG (filled, left panels), anti-S100A13 antibody (line, left panels), control rat IgG (filled, right panels), or anti-human podoplanin (NZ-1, line, right panels) followed by Alexa Flour 488-conjugated anti-mouse IgG.
(TIF)

S6 Fig Immunohistochemistry for S100A13 and smooth muscle actin in the femoral artery injured by FeCl_3_. Frozen-thawed sections of the mouse femoral artery injured by FeCl_3_ were incubated with anti-smooth muscle actin (SMA) antibody (A), anti-S100A13 (B) followed by visualization using anti-goat IgG Alexa Flour 546 and anti-rabbit IgG Alexa Flour 488, respectively. A and B were merged (C). The phage contrast image are shown in E. Nuclei were counter-stained by DAPI (F).
(TIF)

S7 Fig Immobilized S100A13 did not increase thrombus formation under flow when immobilized with collagen in CLEC-2-deficient blood. A) Wild type murine whole blood (WT, i and ii) or CLEC-2-deficient murine whole blood (KO, iii and iv) stained with DiOC_6_ was perfused into capillaries with collagen (i and iii) or collagen plus S100A13 (ii and iv) for 5 min at a shear rate of 1500 s^−1^. Adherent platelets were visualized by confocal laser microscopy. B) The z-stack data were quantified. The thrombus volume was expressed as the cIFI per image (404374 μm^2^). The graph illustrates the percentage of the control (wild type whole blood) cIFI ± SE (n = 3–4).
(TIF)
