# Supplementary figures and images for: Vascular Smooth Muscle Cells Stimulate Platelets and Facilitate Thrombus Formation through Platelet CLEC-2: Implications in Atherothrombosis
Source: PLoS One. 2015 Sep 29;10(9):e0139357. doi: 10.1371/journal.pone.0139357 (PMC4587843; doi:10.1371/journal.pone.0139357)

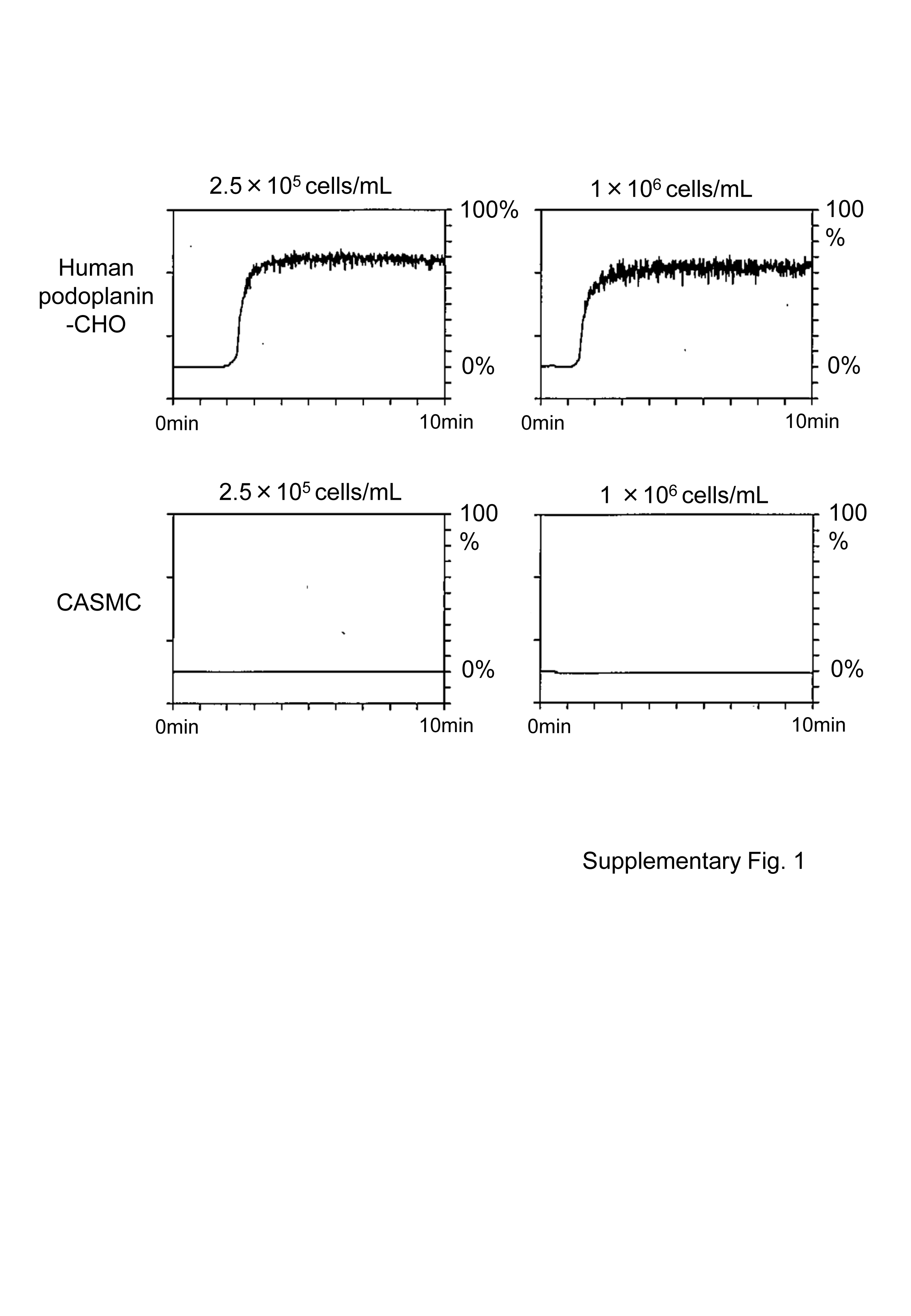

Supplement: S1 Fig — Platelet aggregation by CHO cells transfected with human podoplanin (upper panels) or CASMCs (lower panels) was monitored by light transmission. Final concentrations of the cells were 2.5 × 10^5^ cells/ml (left panels) and 1.0 × 10^6^ cells/ml (right panels). (TIF) [file pone.0139357.s001.tif]

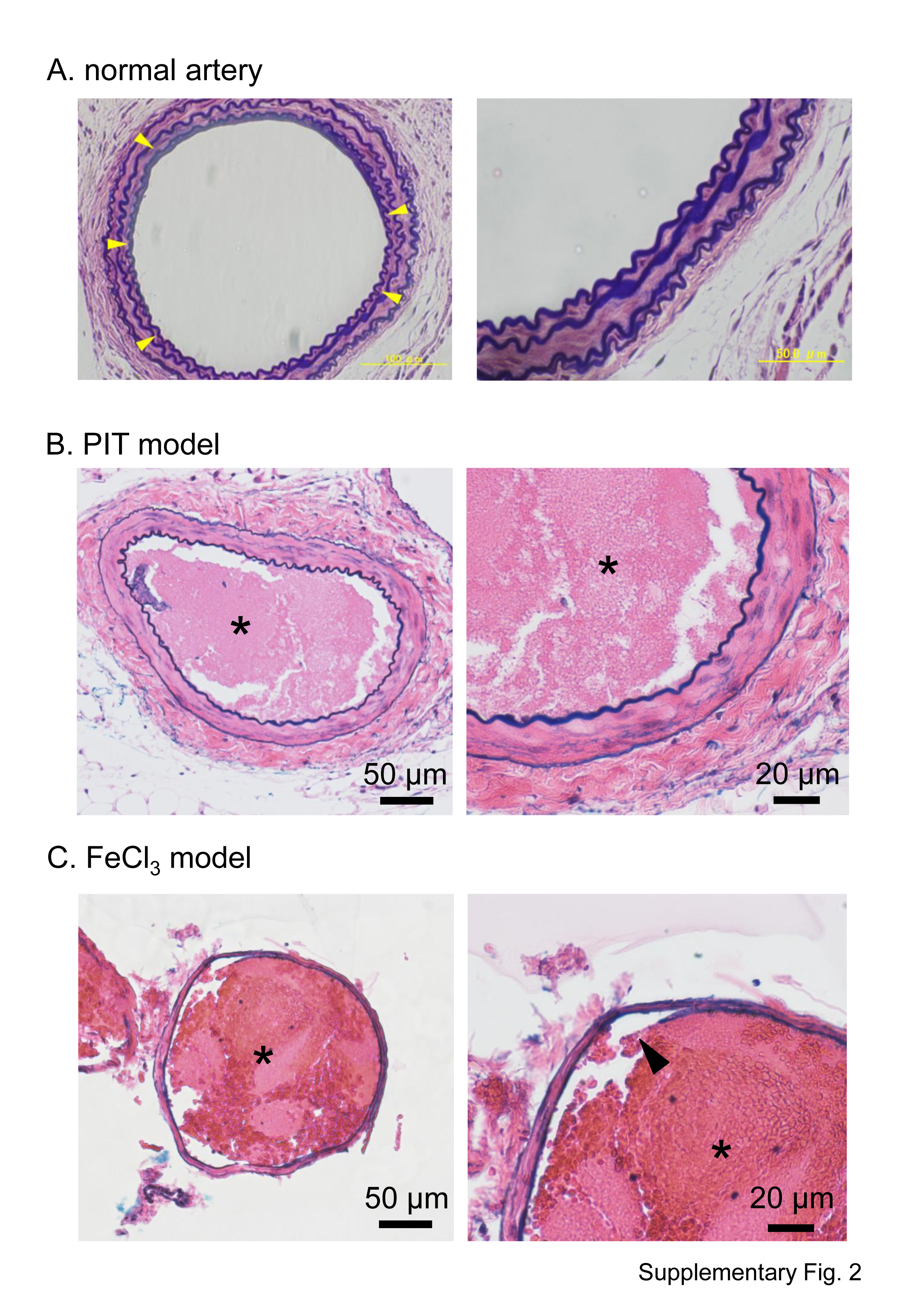

Supplement: S2 Fig — A) Victoria blue-HE staining of non-injured murine femoral artery. Arrowheads indicates internal media lamina. B) Victoria blue-HE staining of photochemically-injured murine femoral artery. C) Victoria blue-HE staining of FeCl3-injured murine femoral artery. Laceration of internal media lamina is indicated by an arrow. The asterisks indicate thrombi. (TIF) [file pone.0139357.s002.tif]

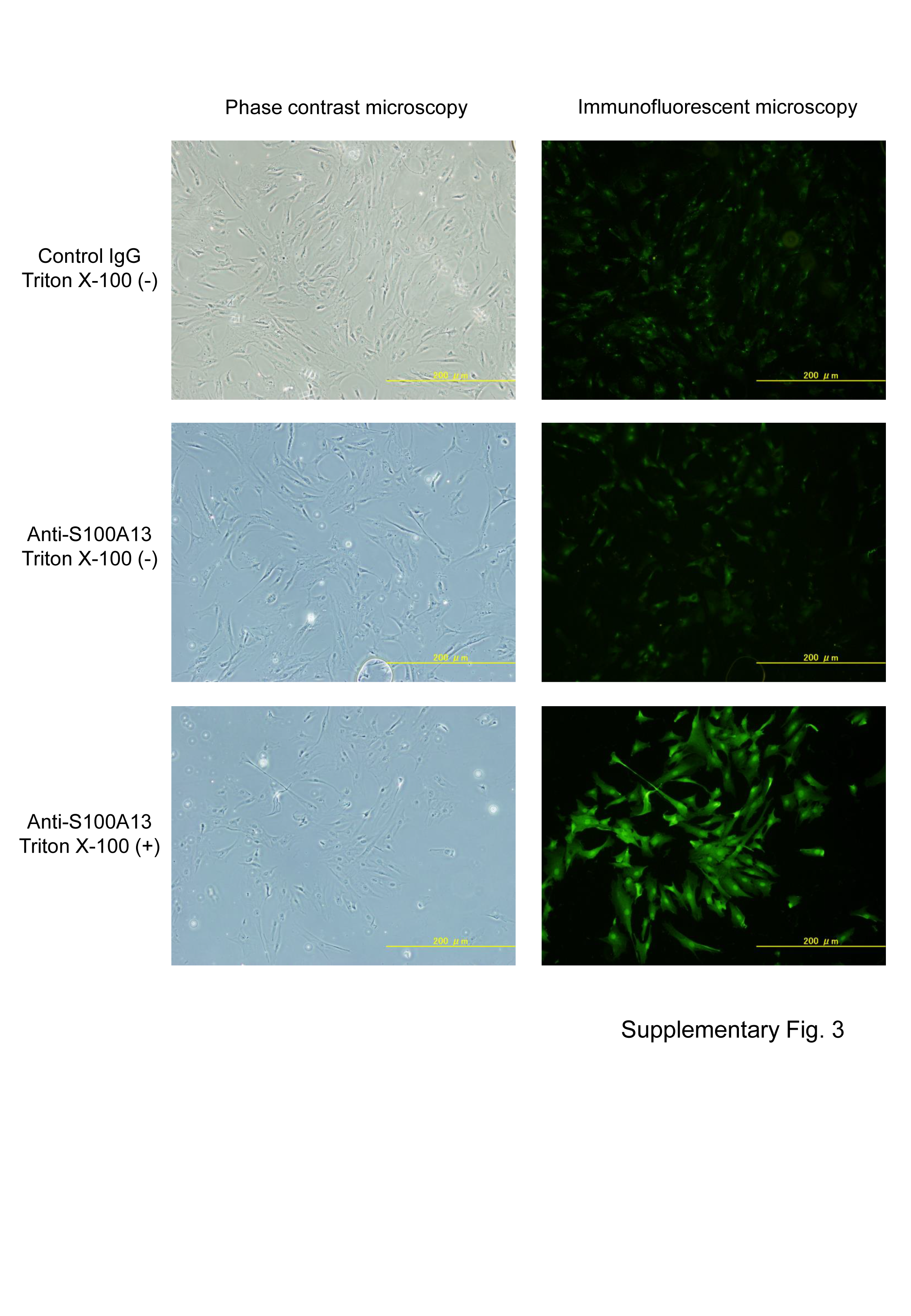

Supplement: S3 Fig — CASMCs cultivated on a 24-well culture dish were fixed and incubated with control mouse IgG (upper panels) or anti-S100A13 antibody (63Y) (middle and lower panels), followed by anti-mouse IgG labeled with Alexa Fluor 488. Where indicated, cells were treated with 0.1% Triton X-100 before addition of the antibodies (lower panels). Cells were visualized by phase-contrast Microscopy (left panels) or fluorescence microscopy (right panels). (TIF) [file pone.0139357.s003.tif]

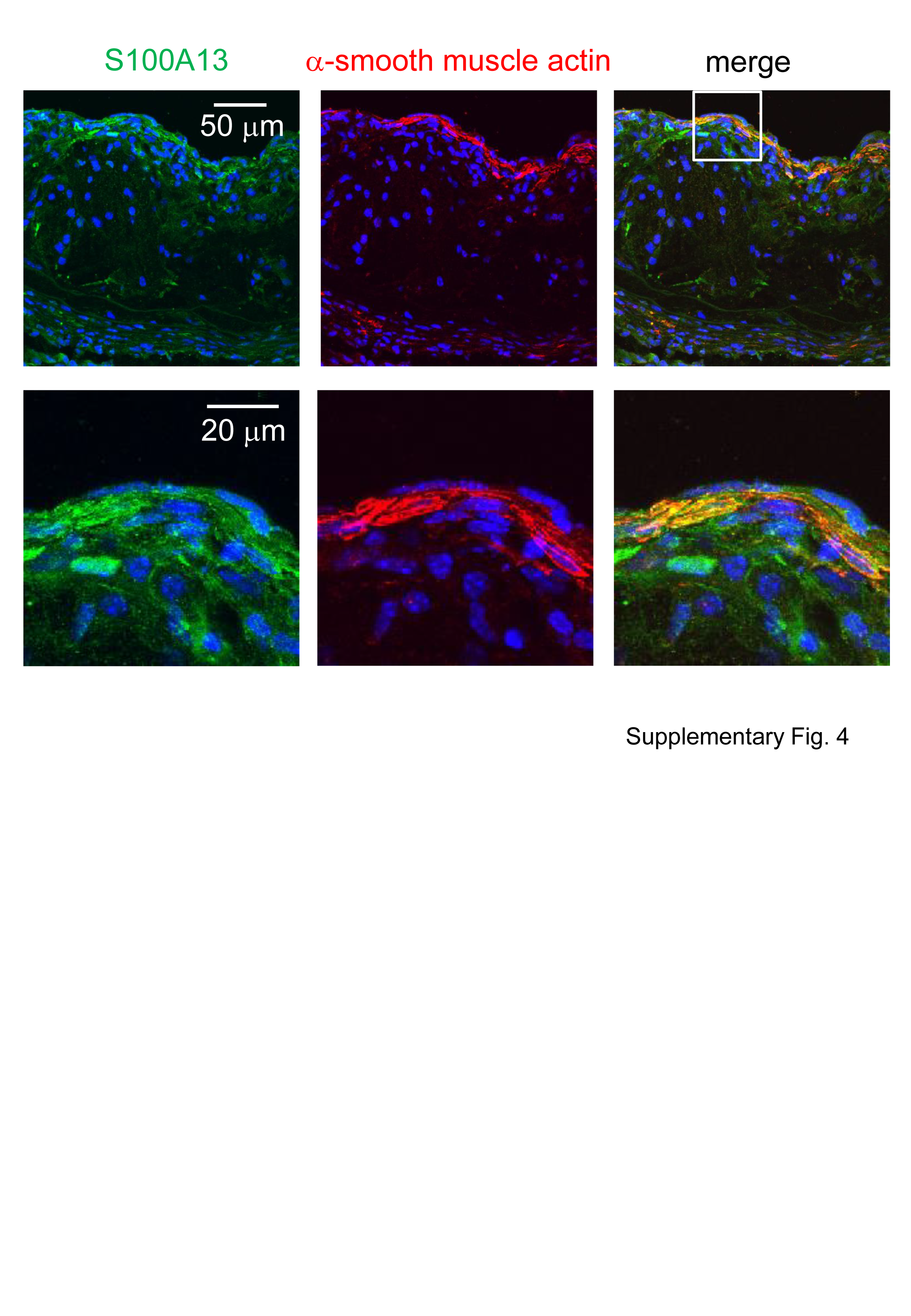

Supplement: S4 Fig — Frozen-thawed sections of the mouse abdominal aorta from ApoE-deficient mice fed a high fat diet were incubated with goat anti-α smooth muscle actin (left panels) or rabbit anti-S100A13 antibody (middle panels), followed by anti-rabbit IgG-Alexa Fluor 488 and anti-goat IgG-Alexa Fluor 597, respectively. Right panels are merged images of left and middle panels. Lower panels are magnified images of the square area from upper panels. (TIF) [file pone.0139357.s004.tif]

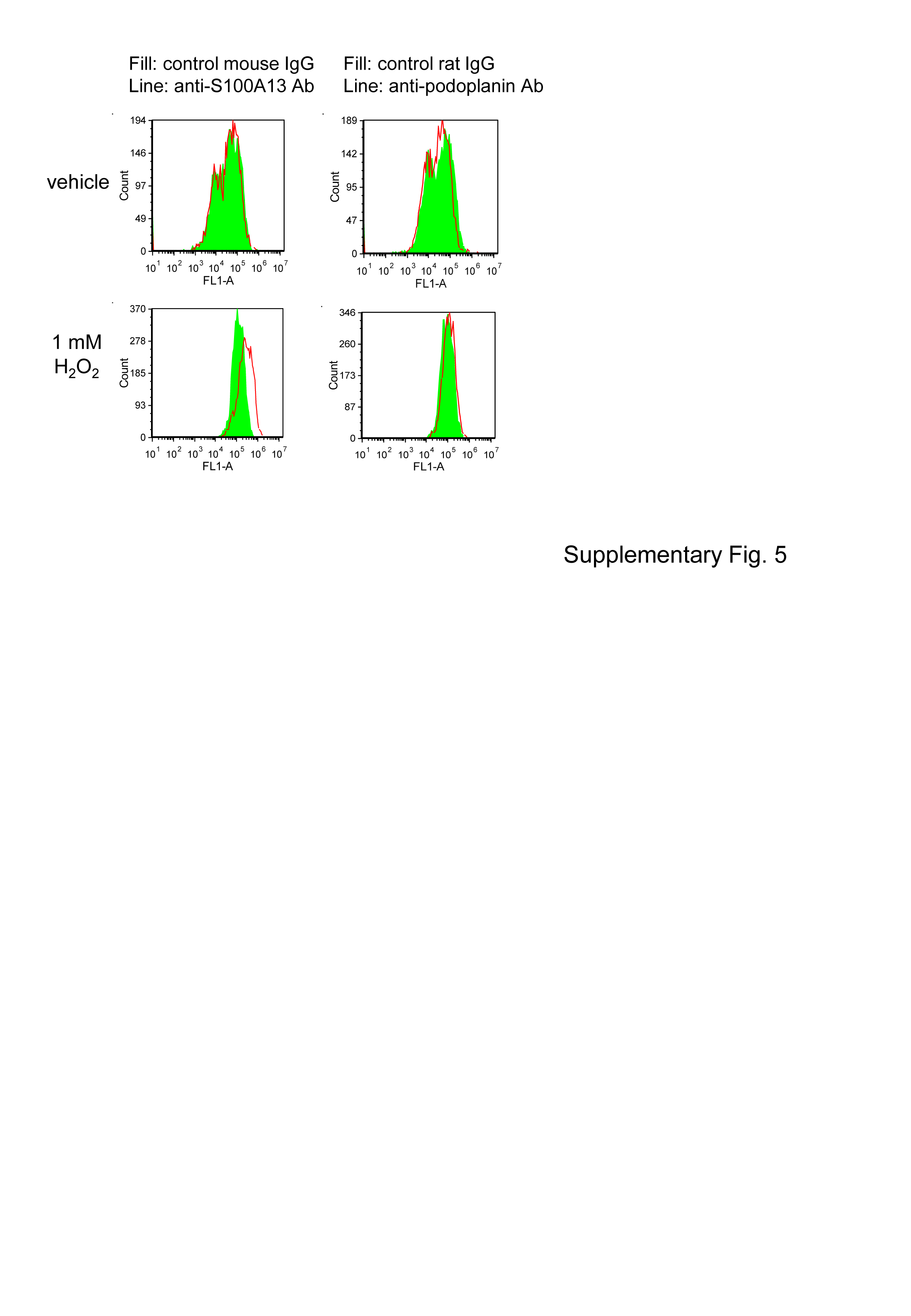

Supplement: S5 Fig — Surface expression of endogenous S100A13 or podoplanin was analyzed by flow cytometry. CASMCs pretreated with vehicle (upper panels) or 1 mM H2O2 (lower panels) were incubated with control mouse IgG (filled, left panels), anti-S100A13 antibody (line, left panels), control rat IgG (filled, right panels), or anti-human podoplanin (NZ-1, line, right panels) followed by Alexa Flour 488-conjugated anti-mouse IgG. (TIF) [file pone.0139357.s005.tif]

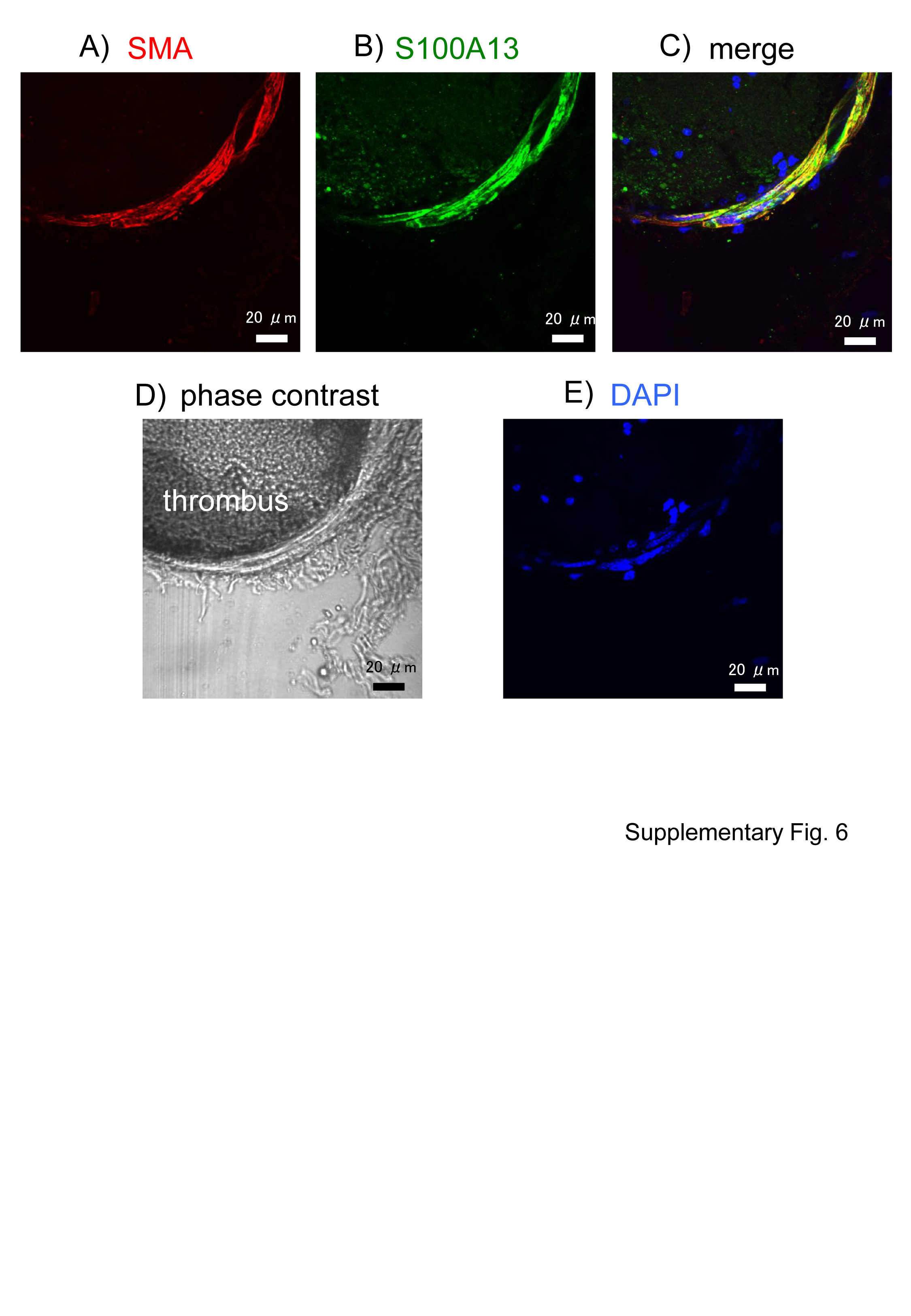

Supplement: S6 Fig — Frozen-thawed sections of the mouse femoral artery injured by FeCl3 were incubated with anti-smooth muscle actin (SMA) antibody (A), anti-S100A13 (B) followed by visualization using anti-goat IgG Alexa Flour 546 and anti-rabbit IgG Alexa Flour 488, respectively. A and B were merged (C). The phage contrast image are shown in E. Nuclei were counter-stained by DAPI (F). (TIF) [file pone.0139357.s006.tif]

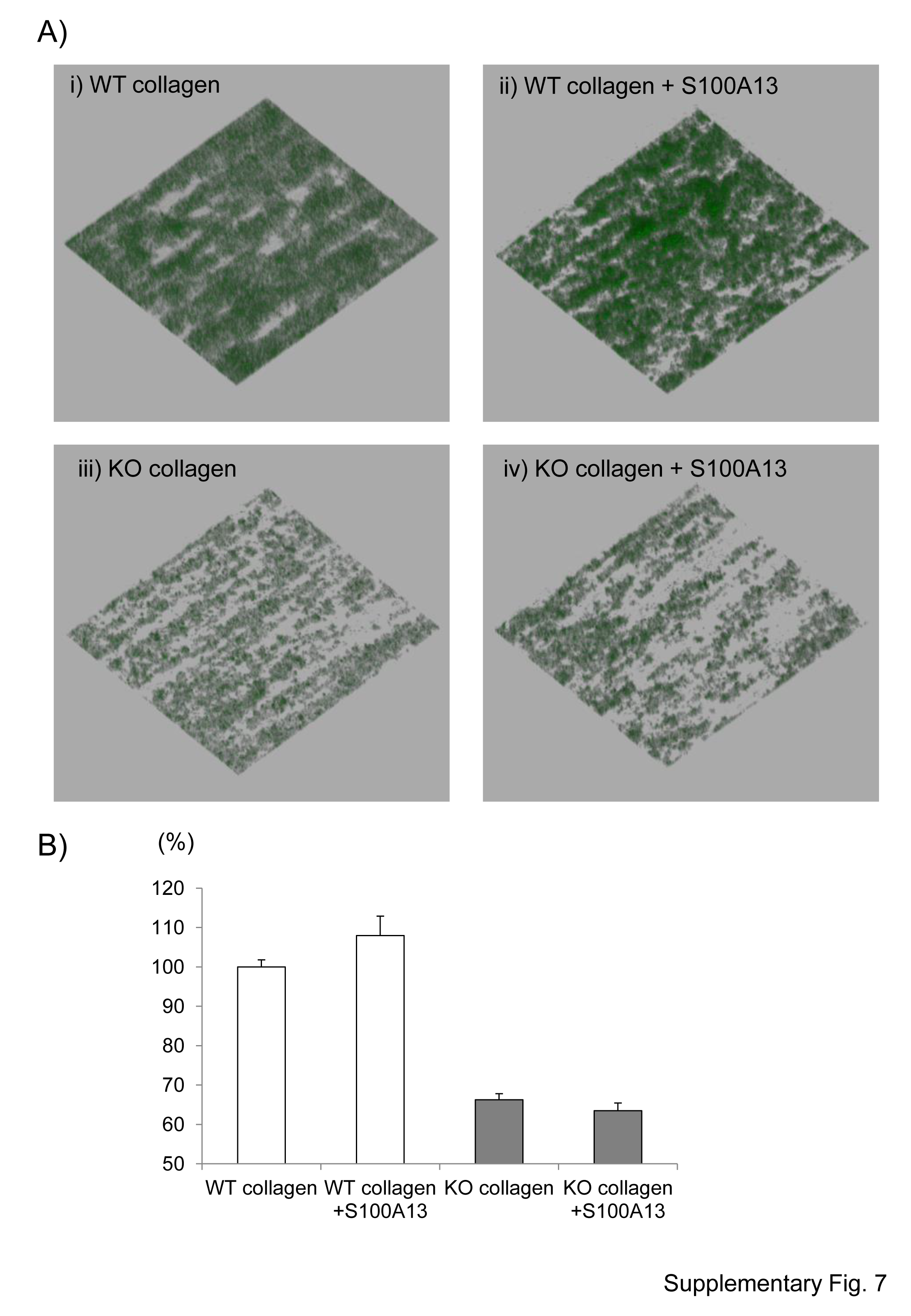

Supplement: S7 Fig — A) Wild type murine whole blood (WT, i and ii) or CLEC-2-deficient murine whole blood (KO, iii and iv) stained with DiOC6 was perfused into capillaries with collagen (i and iii) or collagen plus S100A13 (ii and iv) for 5 min at a shear rate of 1500 s−1. Adherent platelets were visualized by confocal laser microscopy. B) The z-stack data were quantified. The thrombus volume was expressed as the cIFI per image (404374 μm2). The graph illustrates the percentage of the control (wild type whole blood) cIFI ± SE (n = 3–4). (TIF) [file pone.0139357.s007.tif]
